# Nuclear Motility in Glioma Cells Reveals a Cell-Line Dependent Role of Various Cytoskeletal Components

**DOI:** 10.1371/journal.pone.0093431

**Published:** 2014-04-01

**Authors:** Alexa Kiss, Peter Horvath, Andrea Rothballer, Ulrike Kutay, Gabor Csucs

**Affiliations:** 1 Institute of Biochemistry, ETH Zurich, Zurich, Switzerland; 2 Light Microscopy and Screening Centre, ETH Zurich, Zurich, Switzerland; University of Zurich, Switzerland

## Abstract

Nuclear migration is a general term for the movement of the nucleus towards a specific site in the cell. These movements are involved in a number of fundamental biological processes, such as fertilization, cell division, and embryonic development. Despite of its importance, the mechanism of nuclear migration is still poorly understood in mammalian cells. In order to shed light on the mechanical processes underlying nuclear movements, we adapted a micro-patterning based assay. C6 rat and U87 human glioma cells seeded on fibronectin patterns - thereby forced into a bipolar morphology - displayed oscillatory movements of the nucleus or the whole cell, respectively. We found that both the actomyosin system and microtubules are involved in the nuclear/cellular movements of both cell lines, but their contributions are cell-/migration-type specific. Dynein activity was necessary for nuclear migration of C6 cells but active myosin-II was dispensable. On the other hand, coupled nuclear and cellular movements of U87 cells were driven by actomyosin contraction. We explain these cell-line dependent effects by the intrinsic differences in the overall mechanical tension due to the various cytoskeletal elements inside the cell. Our observations showed that the movements of the nucleus and the centrosome are strongly correlated and display large variation, indicating a tight but flexible coupling between them. The data also indicate that the forces responsible for nuclear movements are not acting directly via the centrosome. Based on our observations, we propose a new model for nuclear oscillations in C6 cells in which dynein and microtubule dynamics are the main drivers of nuclear movements. This mechanism is similar to the meiotic nuclear oscillations of *Schizosaccharomyces pombe* and may be evolutionary conserved.

## Introduction

The nucleus is an organelle of central importance in eukaryotic cells. Establishment of a specific position of the nucleus within the cell contributes to key biological processes [1]. Nuclear movements have been described throughout eukaryotes, however their actual functions show remarkable variations. For example, the migration of the nucleus to the bud neck in *Saccharomyces cerevisiae* is required for the proper distribution of the genetic material to the daughter cell [2], [3]. During *Schizosaccharomyces pombe* meiosis, the nucleus performs oscillatory movements [4], which facilitate the recombination of meiotic chromosomes [5]. Further observations in *Caenorhabditis elegans* [6] and *Drosophila melanogaster* [7], [8] embryos have revealed the importance of nuclear migration processes in the development of metazoan organisms.

In contrast to the previous examples, nuclear positioning in vertebrates is poorly characterized and understood. Although recent data indicate that nuclear migration is present in most pseudostratified tissues [9], [10], the best-described *in vivo* example is the developing vertebrate nervous system. Nuclei of neural progenitor cells display a characteristic oscillatory movement known as interkinetic nuclear migration (IKNM) between the apical and basal surface of the neuroepithelium [11], [12]. This type of nuclear migration is cell cycle-dependent, and contributes to neurogenesis [13], [14]. Another type of nuclear migration process is known as nucleokinesis, during which the newly generated neurons leave the neuroepithelium, and migrate distances of several cell lengths along glial fibers to reach their final position in the cortex [15], [16]. As disorders of the neuronal migration pathway lead to neurological diseases, these processes are extensively studied [17], [18].

In the systems investigated so far, nuclear movements are mainly coordinated and powered by cytoskeletal filaments and molecular motors, although mechanical properties of the cytosol may play a role as well. Currently, microtubules and their associated motor proteins are thought to be the main drivers of nuclear migration in most of the studied model systems [19]–[22], but there are also indications of myosin, actin [15], [23], [24], or intermediate filament [25] involvement. The functions of these different cytoskeletal elements in nuclear positioning are thought to be rather competitive or cooperative than mutually exclusive [26]. Nuclear movements, but also the anchorage of the nucleus in a specific position require a linkage between the nucleus and the cytoskeleton [24], [27], [28]. Such connections are mainly mediated via the linker of nucleoskeleton and cytoskeleton (LINC) complexes, evolutionary conserved protein assemblies in the nuclear envelope [29]. The importance of LINC-complex proteins in nuclear positioning, nuclear anchorage and developmental or diseased states has been shown throughout the eukaryotes [30]–[34].

Despite the large number of identified molecular players, the exact biophysical mechanisms of nuclear positioning in mammalian cells are still largely unresolved, and difficult to assess experimentally. *In vivo* studies are often hampered by the fact that most nuclear migration defects lead to embryonic lethality [35]–[38]. *In vitro* nuclear migration experiments are mainly performed using brain slices, dissociated neuronal cultures or wound-healing assays [18], [24], [39], [40]. However, cell-autonomous features of nuclear movements cannot be fully dissected and analyzed in these systems, as the neighboring cells - via cell-cell contacts and/or signaling - represent a constant source of “noise”. Another difficulty in studying nuclear migration under standard cell culture conditions is that nuclear movements in mammalian cells are usually coupled to cell migration, and it is experimentally challenging to separate these two processes without the presence of cell-cell contacts [41], [42]. A novel experimental approach to this problem was introduced by Szabo et al. [43] who have described a new phenomenon, “auto-reverse nuclear migration” in glioma and primary fibroblast cells. They applied micro-contact printing to force cells in an elongated morphology where the intrinsic migratory behavior of the cells decreased, and an oscillatory movement of the nucleus - phenotypically similar to the movements typical for IKNM [44] - was initiated. Despite the advantages offered by this system, the exact mechanical model behind the movement is still unclear, especially in terms of the relative importance of the various cytoskeletal elements [45]. In addition, the exact role of the centrosome in the process of nuclear migration is also disputed, as it was reported both lagging behind the nucleus and leading it [43], [46], [47].

Thus, the aim of this study was to solve the controversy and get a better understanding of mechanical processes underlying nuclear motility. Hence, we applied the above-mentioned assay [43] to study the details of nuclear migration in two different glioma cell lines (rat C6 and human U87). The cell lines were chosen as nuclear oscillations induced by geometrical constrains were previously described in C6 rat glioma cells [43]. In order to extend the assay for other cell types, we investigated the phenomenon also in a human cell line. The U87 human astrocytoma cell line is highly motile in 2D cell culture, and from previous studies [43] it was clear that the presence of nuclear oscillation in this model system is associated with the motility properties of the cells in 2D.

We found that the relative contribution of various cytoskeletal elements (actin and microtubules) and their associated motor proteins are cell-line specific. Our results show that dynein inhibition alone is sufficient to inhibit nuclear movements of C6 glioma cells, but has no effect on their overall cell migration. Furthermore, detailed investigations of the nucleus-centrosome coupling during nuclear movements indicated that the centrosome has no direct force-transmitting role. Based on our results, we suggest a new mechanical model for nuclear oscillations in C6 cells.

## Materials and Methods

### Cell culture and transfection

C6 rat glioma cells were a kind gift of Dr. Emilia Madarasz (Institute of Experimental Medicine, Budapest), U87 human glioma cells were kindly provided by Dr. Harun Said (University of Würzburg) [48], [49]. Cells were cultured in DMEM (Dulbecco's Modified Eagle Medium, Sigma), supplemented with 10% FBS (Fetal Bovine Serum, PAA, Pasching, Austria) and penicillin/streptomycin (PAA) at 37°C, in a humidified atmosphere incubator containing 5% CO2. For generation of centrin-2-GFP stable cell lines, cells were transfected with a pIRESneo3-centrin-2-GFP plasmid (from Yagmur Turgay, ETHZ, IBC) using FuGENE transfection reagent (Roche). The clones were selected and maintained in a G418 (PAA) selection medium for further assays. C6 cells were transiently transfected with a pIRES-puro-EB3-YFP plasmid (from Andrea Rothballer, ETHZ, IBC) using FuGENE reagent (Roche).

### Micro-patterning

PDMS (poly-dimethyl-siloxane) stamps containing an array of 200/5 μm (length/width) rectangles were cleaned by sonication in 70% EtOH for 10 minutes, and dried with pressed air. The patterned surface of the stamp was coated with a mixture of 40 μg/ml fibronectin (Sigma) and 20 μg/ml Alexa594 labeled fibrinogen (Molecular Probes) for 30 minutes, and transferred by micro-contact printing to the substrate (glass coverslips (Merck) or 6-well tissue culture plates (Greiner)). Fluorescently labeled fibrinogen was added to the fibronectin solution in order to visualize the structures. A 0.25 μg/ml solution of poly(L-lysine)-g-poly(ethylene glycol) (PLL-PEG, JenKem) was applied to inhibit cell attachment (for more details see [50]).

### Microscopy

Time lapse images were acquired using the UPLANFLN 10×0.3 NA objective of an Olympus IX 81 inverted microscope equipped with a Hamamatsu Orca camera and an incubator box (EMBL Heidelberg) to maintain 37°C and 5% CO2 for long-term live cell imaging. Images were taken in every 5 minutes by using the Multi-dimensional acquisition application of the Metamorph software (Molecular Devices). For higher resolution imaging the 60×1.42 NA Oil PlanApoN objective was used. Alternatively, immunostained images (107×107×200 nm voxel size) were deconvolved using the Huygens package (Scientific Volume Imaging).

C6 cells expressing YFP-EB3 were imaged with the 63×1.2 NA water objective of a Zeiss Axio Observer Z1 spinning disc microscope equipped with an incubator box (PeCon GmbH). A single plane was captured using the 488 nm laser line, with a sampling interval of 400 ms.

### Cytoskeletal inhibitors

To estimate the contribution of microtubules, actin and cytoskeletal motor proteins their specific inhibitors were applied. Microtubule depolymerization was induced by 10 nM nocodazole (Sigma-Aldrich), whereas microtubules were stabilized by 0.5 nM taxol (Sigma-Aldrich). Actin dynamics was influenced by 100 nM of cytochalasin D (Sigma-Aldrich). Myosin II activity was blocked by 10 μM blebbistatin (-/-, Sigma-Aldrich), whereas dynein was inhibited using 0.5 mM EHNA (erythro-9-(2-hydroxy-3-nonyl)-adenine, Sigma-Aldrich). In these inhibition experiments, C6 and U87 cells were seeded in patterned (stamped) 6-well tissue culture plates (Greiner), and allowed to spread for 3 hours. Inhibitors were added shortly before imaging, and were kept on the cells for the duration of the experiments. Cells were imaged for at least 14 hours in the presence of either a solvent control (DMSO (Sigma-Aldrich) or water) or one of the indicated drugs.

### Immunofluorescence

Cells, grown on coverslips were fixed with 4% paraformaldehyde (Sigma-Aldrich) for 15 minutes at room temperature. After permeabilization with 0.01% Triton X-100 (Sigma-Aldrich) for 5 minutes, non-specific protein binding was blocked by 10% goat serum (Sigma-Aldrich) dissolved in 2% BSA-PBS. Antibody against alpha-tubulin (1∶2000 dilution, mouse, T-6074, Sigma-Aldrich) and coumarin- or Alexa594-coupled phalloidin (1∶250 dilution, Molecular Probes) were used to label microtubules and actin filaments. Alexa Fluor 488 goat anti-rabbit (1∶400 dilution, Molecular Probes) was applied as secondary antibody. Alternatively, cells were fixed with 100% −20°C methanol (Merck) for 8 minutes, and stained with dynein (1∶100 dilution, abcam, ab23905) and alpha-tubulin (1∶1000 dilution, abcam, ab18251) antibodies.

### Data processing

The Manual Tracking plugin of ImageJ was applied to determine the *x-y* positions of the nucleus and the centrosome in the maximum intensity projections of the fluorescent images (necessary due to the high cytoplasmic background of GFP-centrin-2). In order to measure the displacement of cell nuclei on phase contrast images, a custom-written tracking software, the CellTracker was developed in MATLAB (R2012b, The Mathworks, Natick, MA). The tracking process consists of the following steps: 1) Background subtraction in order to remove image inhomogeneity caused by uneven illumination or dirt. 2) Image cross-correlation-based (between consecutive frames) alignment was used to correct possible paraxial stage drifts. 3) Actual tracking using an automated or semi-automated mode. The obtained *x*-*y* coordinates of the nucleus and the centrosome were transferred into a new coordinate system in which the „central position“ of the nuclear displacement along the pattern became the new origin (0,0), and the longer axis of the pattern refers to the “movement axis”. Velocity of the nucleus was calculated from its displacement between two consecutive time frames as 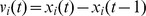, and was smoothed for a 30-minutes interval with a moving average function. Tracked interphase nuclei were classified as „oscillating“, „irregular movement“, „no movement“ by visual observation of the changes in their nuclear position over time (see Figure S2 for details). In order to identify the local maxima and minima of the periodic curves, the built-in findpeaks MATLAB function was used with the following criteria: the peaks needed to be located at least 12 frames away from each other, and have a minimum amplitude of 25 μm. With this constrain we could greatly eliminate the effect of noise.

### Statistical analysis

Statistical analysis was performed in R (R Development Core Team). The selected p-value for significance was p<0.05. To determine significance of the effects of distinct inhibitors on nuclear movements, either the Kruskal-Wallis test or the Wilcoxon test was used. For multiple comparisons, paired Wilcoxon test or Nemenyi-Damico-Wolfe-Dunn test [51] was applied. Correlations between calculated parameters of centrosome and nucleus (positions, speeds, nucleus speed, nucleus-centrosome distance) were characterized by Pearson's correlation coefficients. Cross-correlation analyses of nuclear and cell, or nuclear and centrosome movements were performed in MATLAB, applying the built-in cross-covariance function with normalization.

## Results

### Glioma cells adapt to geometrical constrains, and show nuclear/whole cell oscillations

In our experiments, C6 rat glioma and U87 human glioma cells were attached on 200/5 μm adhesive fibronectin rectangles, based on a previous assay [43]. The dimensionality of these patterns favors single-cell adhesion, thereby enabling analysis of cell-autonomous movements. Furthermore, the small pattern width (5 μm) allows us to consider predominantly one-dimensional/uniaxial displacements.

**Figure 1:**
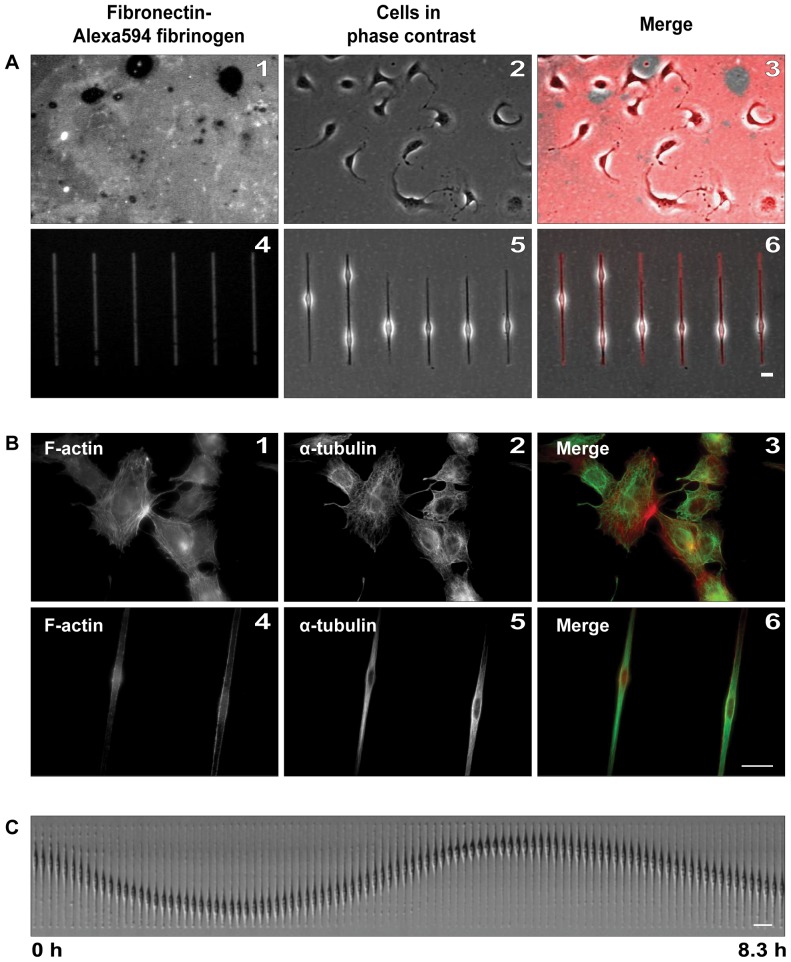
Modified cell shape and cell motility on micro-patterned surfaces. C6 rat glioma cells were plated on fibronectin-Alexa594 fibrinogen either on homogenous (A1–A3) or on patterned (A4–A6, 200/5 μm rectangles) surfaces. The fluorescent protein coat (A1 and A4), phase contrast images of the cells (A2, A5) and the merge of both (A3, A6), are shown. Actin and microtubules were labeled with coumarin-phalloidin (B1 and B4) and alpha-tubulin antibody (B2 and B5), respectively. In the overlays, actin filaments are red; microtubules are green (B3 and B6). C) Phase-contrast kymograph of a single C6 cell showing nuclear oscillation. Scale bars: 20 μm

C6 rat glioma cells seeded on the fibronectin rectangles gained an elongated, bipolar morphology (Figure 1 A, panels 4–6) in contrast to cells attached to a homogenous protein-coated surface (Figure 1 A, panels 1–3). We performed immunofluorescent stainings of various cytoskeletal elements (Figure 1 B and Figure S1), which indicated the adaptation of cytoskeleton to the underlying surface pattern, and the subsequent modification of cell geometry. Microtubules maintained the usual radial arrangement in cells attached to homogenous protein surface (Figure S1, B), but they were aligned parallel to the cells' long axis in cells seeded on patterns (Figure S1, D). In cells plated on non-patterned substrates, actin stress fibers were detectable across the whole cell, but under geometrical confinement these were limited to the cell periphery (Figure S1 A and C). U87 cells possessed similar phenotypes as C6 cells under all conditions (data not shown). Although the typical broad lamellopodial protrusions observed on 2D surfaces were absent in elongated C6 and U87 cells, protrusion-retraction cycles of the leading and trailing edges were detected. The application of micro-patterns clearly changed the motility behavior of the cells in contrast to homogenous surfaces (Movie S1 and Movie S2, Figure 1 C). In order to quantitatively assess these differences - especially in respect to the movements of the cell nucleus - we tracked the nuclei of individual cells using the CellTracker software (see Materials and Methods). After the automated tracking, we classified nuclear movements into 3 phenotypically distinct groups by repeated visual observation and categorization of the obtained trajectories: “oscillation”, “irregular movement” and “no movement” (Figure S2, only U87 cells are shown). According to this classification, 51±2% of C6 cells and in 52±1% of U87 cells displayed oscillatory movements of the nucleus, respectively.

### Contributions of cytoskeletal elements and motor proteins to oscillations vary with the cell line

**Figure 2:**
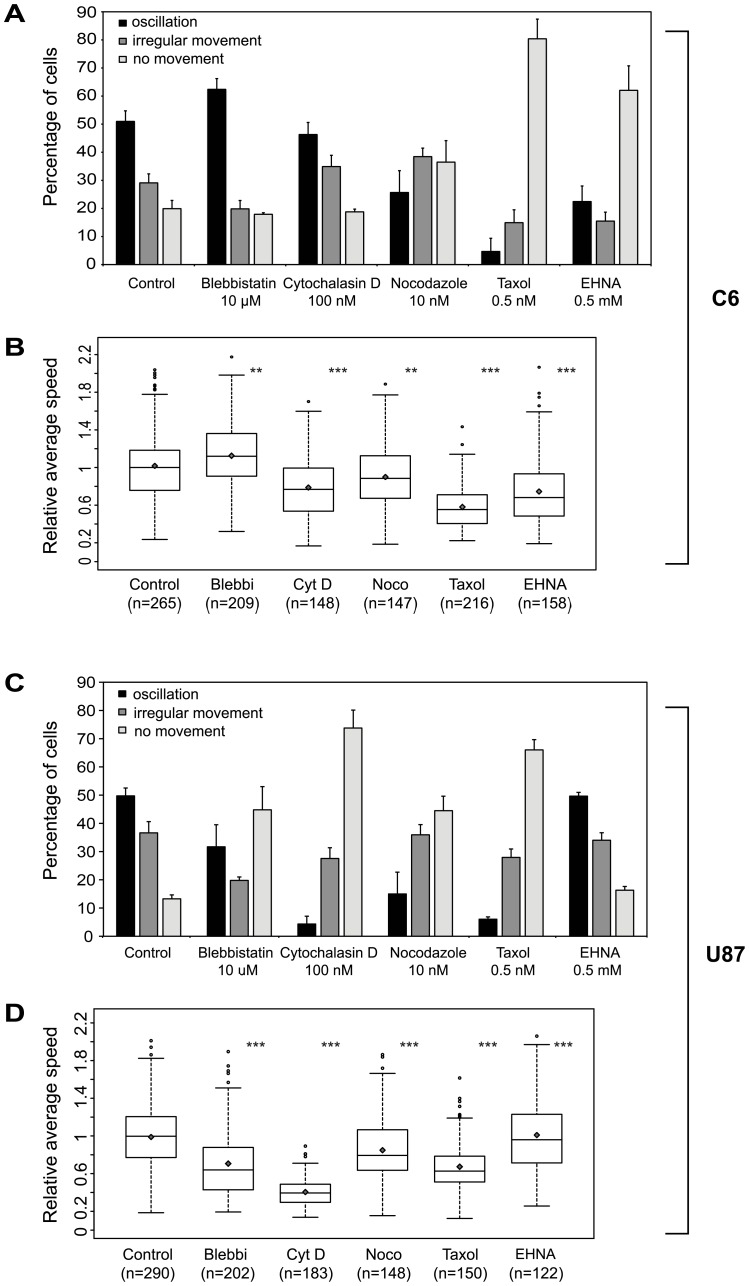
Cell-line dependent effects of actin and microtubule inhibitors on nuclear motility in C6 and U87 cells. Cells were imaged for at least 14(solvent control) or various cell-permeable cytoskeletal drugs. A–B: C6 cells, C–D: U87 cells (A and C) Proportion of cells within the different motility subgroups^†^ upon inhibitor treatments. Error bars indicate mean+SE. (B and D) Average nuclear speeds in the two cell lines. Diamonds mark mean values, empty circles represent outliers. Data of at least three independent experiments is shown. Statistical analysis was performed using Kruskal-Wallis test. Significance codes: ^*^: p<0.05, ^**^: p<0.01, ^***^: p<0.001 ^†^: explanation of the three migration types is discussed in Figure S3.

In order to identify the cytoskeletal components contributing to nuclear (and the coupled cellular) movements, several experiments were performed using various inhibitors of actin or microtubule dynamics, or of cytoskeleton-associated motor proteins. In these experiments, micro-patterned C6 or U87 cells were imaged overnight in the presence of either DMSO (solvent control), or one of the indicated drugs (kymographs of example control and inhibitor-treated C6 cells are shown in Figure S4). The general inhibitor efficacy was assessed by immunofluorescent stainings of cytoskeletal components (data not shown). We quantified the inhibitor effects as the percentage of cells falling into one of the three previously established motility categories (Figure S2), and by analyzing the changes in normalized speeds of the total cell population (Figure 2 B and D).

Inhibition of microtubule polymerization (nocodazole) or induction of microtubule stabilization (taxol) strongly decreased the percentage of oscillating nuclei/cells in both cell lines (Figure 2 A and C: percentage (mean ± SE) of oscillating cells: C6: 26±8% of nocodazole- and 5±4% of taxol-treated cells vs. 51±4% in control; U87: 15±8% with nocodazole and 6±1% with taxol vs. 49±3% in control). Actin perturbation by cytochalasin D [52] also interfered with cellular and nuclear movements in both cell lines, but to a different extent. The average cell population speed and the proportion of oscillating cells were severely reduced in U87 cells (Figure 2 D: median speed ± IQR: 22±11 μm/h vs. 50±30 μm/h in control, Figure 2 C: 5±2% oscillating cells upon cytochalasin treatment), opposed to C6 cells (Figure 2 B: median speed ± IQR: 23±14 μm/h with cytochalasin D vs. 29±15 μm/h in control cells, Figure 2 A: 46±4% oscillating cells upon treatment). In addition to the drop in the number of oscillating cells, the proportion of irregularly moving C6 cells increased (35±1% vs. 29±3% of control cells), which may account for the milder cellular velocity changes observed in the total C6 cell population. Taken together, these results suggest that both the microtubule and actin cytoskeletons play a role in oscillatory movements, although U87 cells seem to be more sensitive to actin depolymerization than C6 cells.

In addition, the two cell lines responded differently to the applied motor protein inhibitors. Blebbistatin (an inhibitor of non-muscle myosin II [53]) repressed oscillations of U87 cells, but increased the percentage of oscillating C6 cells (Figure 2 A and C; 31±8% vs. 49±3% of U87 cells and 68±7% vs. 51±4% of C6 cells). Inhibition of cytoplasmic dynein by EHNA [54] had diverse effects on nuclear/cell motility of the investigated cell lines. In C6 cells, EHNA severely perturbed nuclear movements (Figure 2 A and B, 22±6% of cells were oscillating; median speed ± IQR: 11±8 μm/h vs. 29±15 μm/h in control), whereas this drug had lower impact on U87 cells (Figure 2 B and D, 50±1% oscillating cells, median speed ± IQR: 39±18 μm/h vs. 48±30 μm/h in control). An alternative dynein inhibitor, HPI-4 [55] had similar effects on the oscillatory properties (data not shown). These diverse responses to the various motor activity perturbations indicate that the observed oscillatory movements may rely on different mechanisms in these cell lines.

### Inhibitors of cytoskeletal and motor proteins affect oscillating cells

The applied inhibitors influenced the fraction of oscillating cells and the migration speed of the whole C6 and U87 cell population. To extend our analysis beyond these general effects, we compared the speed and oscillatory properties (period length, amplitude) of the cells previously classified into the “oscillating” subpopulation (black bars in Figure 2 A and C, Table S1). The drugs showed specific effects also in this analysis. With the exception of taxol and EHNA, the average speed of nuclei was similar to the control in C6 cells, but the amplitude and period length of oscillations increased upon inhibitor treatments. In U87 cells, blebbistatin and cytochalasin D strongly reduced the speed of oscillating cells, and increased the period lengths, however the amplitude of oscillations remained close to that of the control cells (Table S1).

These results show that C6 and U87 cells are different in their oscillatory properties, as nuclear oscillations of C6 cells are slower, but have higher period lengths and half-peak amplitudes than the coupled nuclear and cellular oscillations of U87 cells (Table S1).

### Effects of dynein inhibition are dependent on the cellular morphology in C6 cells

As shown above, the motility of geometrically constrained C6 and U87 cells strikingly differs and perturbations of non-muscle myosin II or dynein lead to distinct responses in the two cell lines. Through combined inhibition experiments we aimed to get a better understanding of the underlying mechanisms. Consequently, we treated C6 and U87 cells with blebbistatin, EHNA or with their combination (Figure S5). To control the effect of cellular morphology, cells were plated both on micro-patterns (1D case) and on homogenously coated fibronectin surfaces (2D case, Figure S5). In cells seeded on patterns, the results obtained with single drugs were in agreement with our previous observations in both cell lines (Figure 2), but the combination of inhibitors slightly increased the proportion of oscillating C6 cells (Figure S5 A, mean ± SE: 28±7%) compared to EHNA alone (mean ± SE: 24±3%). In U87 cells the combination decreased this percentage further (mean ± SE: 28±5%) compared to the treatment with blebbistatin alone (mean ± SE: 34±3%).

In the 2D case (migration without geometrical constrains), U87 cells showed a very similar behavior to the 1D scenario regarding their speed of movement (Figure S5 B and C, left panel). Intriguingly, in C6 cells neither myosin (blebbistatin: median speed ± IQR: 21±9 μm/h), nor dynein inhibition affected cell migration speeds significantly (EHNA: median speed ± IQR: 21±8 μm/h vs. 22±9 μm/h in control). Only the combined inhibitor treatment reduced the measured speeds (16±9 μm/h). In summary, these results indicate, that the effects of myosin and dynein inhibition on cell migration are cell geometry-dependent in C6 cells, but not in U87 cells.

### Non-muscle myosin II influences the coupling between cellular and nuclear movements in geometrically confined cells

**Figure 3:**
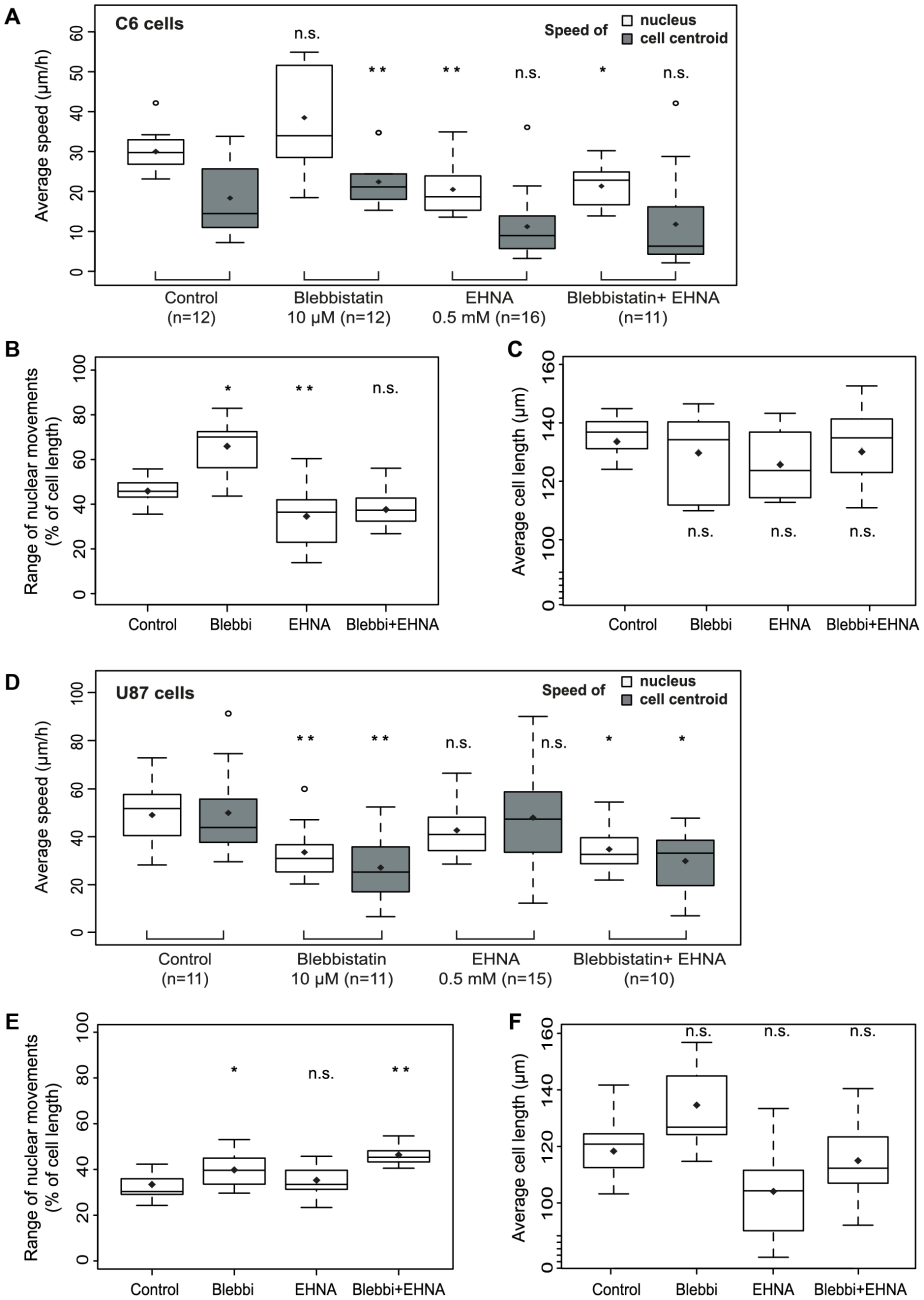
Myosin-II and dynein inhibition have distinct effects on nuclear vs. cellular movements in C6 cells. Cell extensions and nuclei of selected oscillating cells were manually tracked upon treatment with blebbistatin, EHNA, or their combination. (A and D) Average nuclear (centroids) and cell centroid speeds in control (DMSO) and treated C6 and U87 cells. (B and E) Range of nuclear movements: the difference between the maximum and the minimum relative nuclear positions in oscillating C6 (B) and U87 (E) cells upon inhibitor treatments (C and F) Average cell length of C6 (C) and U87 cells (F) compared to control population. Mean values are marked by diamonds, empty circles represent outliers. Significance codes: ^*^: p<0.05, ^**^: p<0.01

Despite of the geometrical constrains, the movements of the nucleus and the cell - especially in U87 cells - are still connected (Figure S3). Therefore, while assessing the effects of certain inhibitors on nuclear oscillations, their influence on nuclear vs. cell motility, and their impact on cell length were also investigated. Separate tracking of the nucleus and the cell extensions and calculation of the relative nuclear movements (Figure S3) enables isolated investigation of nuclear and overall cell migration. For this analysis, we selected oscillating cells based on their trajectories (displacement along the pattern, Figure S3), and manually tracked their cell extensions and their nuclei. Speeds of nuclei and geometrical cell centroids (Figure 3 A and D), the range of nuclear movements (Figure 3 B and E), and average cell length (Figure 3 C and F) were calculated.

Treatment of C6 cells with blebbistatin slightly enhanced the speed of nucleus (median speed ± IQR: 34±20 μm/h vs. 30±6 μm/h in control cells), and the range of nuclear movements, whereas EHNA decreased the speed of oscillating nuclei (Figure 3 A, median speed ± IQR: 18±8 μm/h). Blebbistatin increased the speed of the cell centroid significantly (median speed ± IQR: 21±5 μm/h vs. 14±15 μm/h in control cells), whereas EHNA caused only slight changes (median speed ± IQR: 10±9 μm/h). In U87 cells, nuclear and cellular movements showed a strong correlation, so it is not surprising that inhibitors affected them in a similar fashion. The speeds of the nuclei (Figure 3 B, median speed ± IQR: 38±12 μm/h vs. 60±18 μm/h in control) and the cell centroids (median speed ± IQR: 32±19 μm/h vs. 52±19 μm/h in control) were decreased significantly by myosin II inhibition, but not by blocking dynein activity (nucleus: median speed ± IQR: 49±15 μm/h; cell median speed ± IQR: 55±27 μm/h, Figure 3D). The range of nuclear movements were slightly increased by blebbistatin, and decreased by EHNA in both cell lines (Figure 3 B and E). None of the inhibitors caused a significant change in cell length (Figure 3 C and F).

Additionally, we performed cross-correlation analysis of the positions of the nucleus and the cell centroid over time (Figure S7 A and B) as a function of inhibitor treatment. The effects of inhibitors were characterized by determining the maximal cross-correlation values and the lag times corresponding to these maxima. In C6 cells, myosin inhibition slightly reduced the lag time (mean ± SE: 5±5 frames vs. 13±8 frames in control) and increased the correlation between nuclear and cellular movements (Figure S7 C, cross-correlation maximum (mean ± SE): 0.77±0.07 vs. 0.73±0.1 in control). This strengthened coordination between cell and nuclear movements might be connected to the slightly increased cellular movements (Figure 3). In oscillating U87 cells, the nucleus-cell movement coupling was reduced (Figure S7 C, cross-correlation maximum (mean ± SE): 0.78±0.1 vs. 0.85±0.08 in control cells) upon blebbistatin treatment. Notably, myosin inhibition decreased the motility of cell extensions and at the same time the nucleus showed slow oscillatory movements (Figure S6), phenotypically very similar to C6 cells. Dynein inhibition did not seem to affect the correlation between the movement of the nucleus and the cell centroid in any of the cell lines (cross-correlation maximum (mean ± SE): 0.72±0.05 in C6 cells, 0.85±0.06 in U87 cells). The combination of dynein and myosin inhibitors decreased nucleus-cell coupling in both cell lines (cross-correlation maximum (mean ± SE): 0.69±0.04 in C6 cells, 0.79±0.05 in U87 cells). In summary, the data suggests that in U87 cells coupled oscillatory movements of the nucleus and cell require myosin, but not dynein activity, whereas in C6 cells reduced myosin activity increases the coordination of nucleus-cell movements.

### The centrosome moves with the nucleus, but lags behind during oscillations

**Figure 4:**
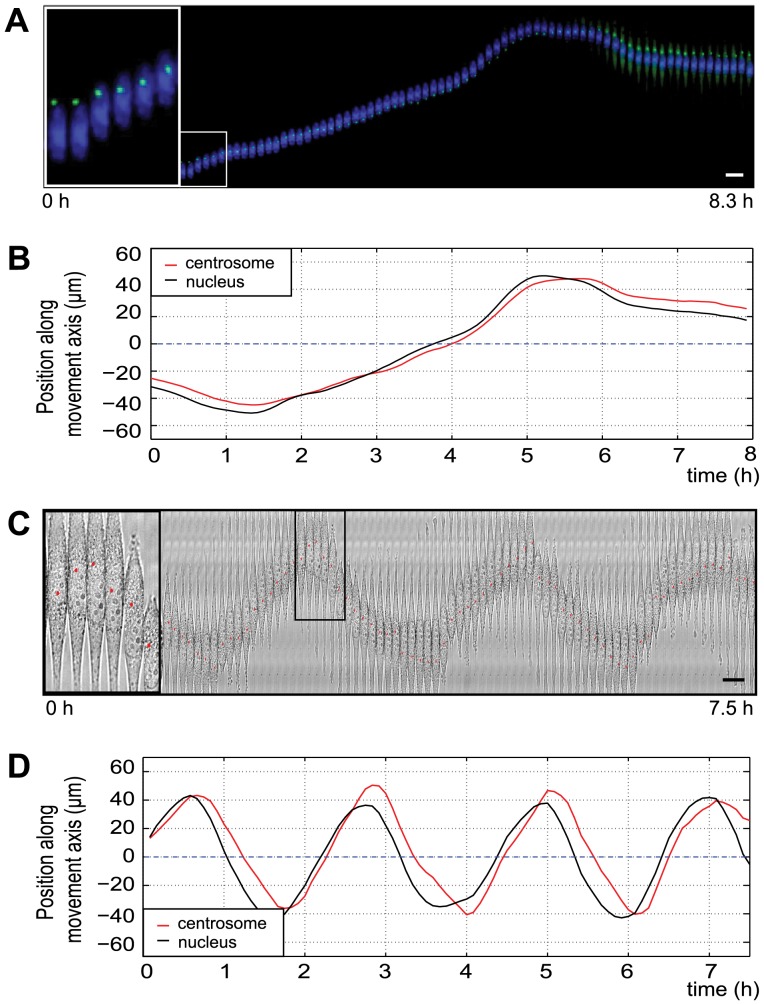
Centrosome and nucleus positioning within oscillating C6 and U87 cells. (A) Kymograph of a GFP-centrin-2 expressing C6 cell, moving along the fibronectin pattern (Hoechst staining: blue, GFP-centrin-2: green). (C) Overlay of a phase contrast image and the position of the centrosome (red dot, inset) in a U87 cell. The centrosome position was determined by manual tracking of the GFP-centrin-2 signal (not shown). (B and D) Nucleus and centrosome positions of the previously presented cells plotted over time. Note that the centrosome (red lines) is lagging behind the nucleus (black lines). Scale bars: 20 μm.

The previous results clearly indicate the involvement of microtubules in nuclear motility. In mammalian cells the centrosome plays a major role in microtubule organization, hence we wanted to explore whether it is directly involved in the nuclear movements especially as previous results involving also other model systems report a very controversial role of the centrosome in nuclear and cellular migration [40], [43], [56]–[58]. To elucidate the nucleus-centrosome relationship, we performed high-resolution live-cell imaging experiments using U87 and C6 cell lines stably expressing GFP-centrin-2. Representative kymographs and videos of oscillating C6- and U87-centrin-2 cells are shown in Figure 4 A and C and Movies S3 and S4, respectively. Corresponding positional plots (Figure 4 B and D) - displaying the changes in nucleus and centrosome locations over time within the same cells – indicate coupled movements of the nucleus and the centrosome in both cell lines.

**Figure 5:**
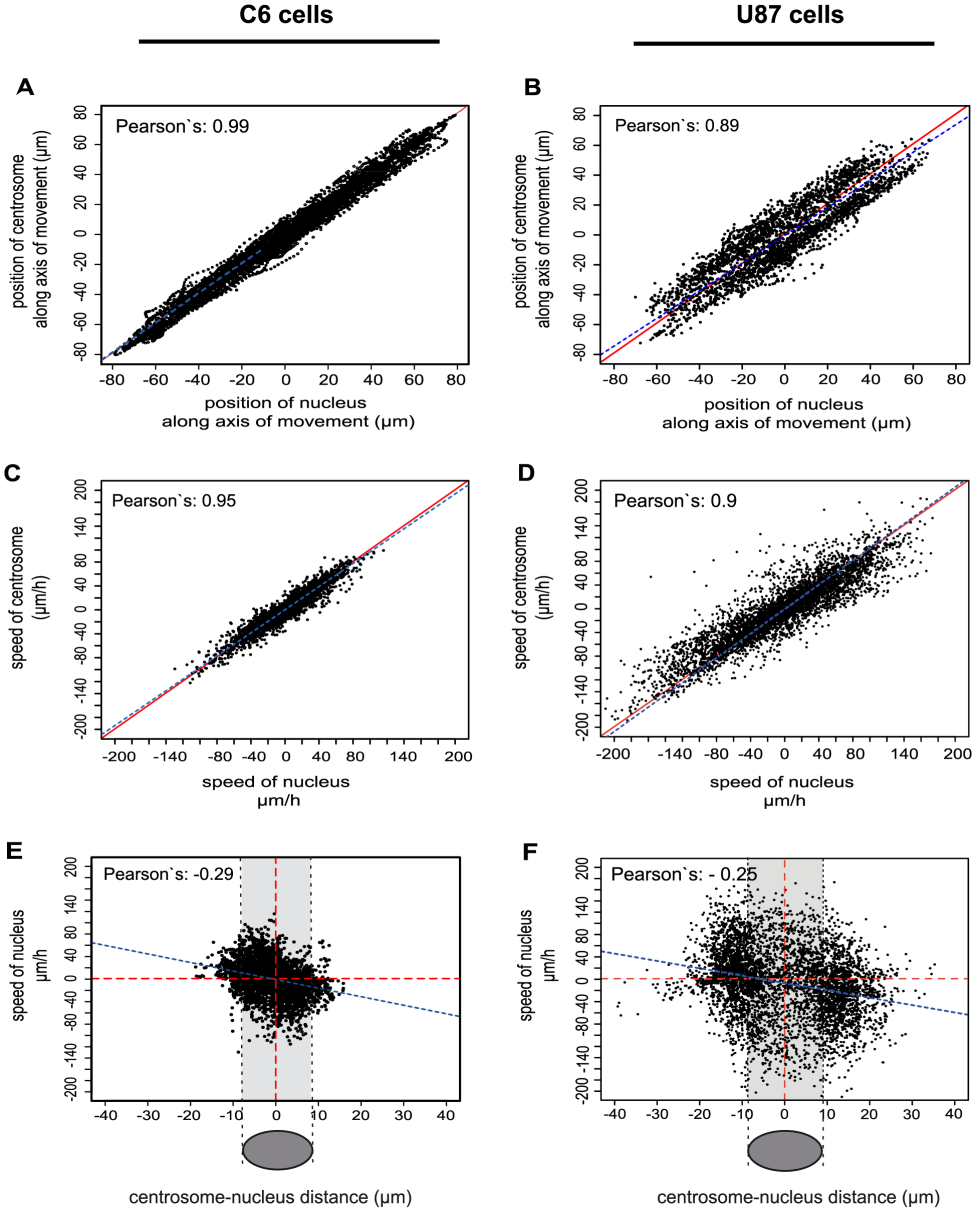
Movements of the nucleus and centrosome are strongly correlated in C6 and U87 cells. Data are shown for C6 (A, C, E) and U87 (B, D, F) cells. Correlations of the nucleus and centrosome coordinates (A and B), movement speed (C and D) and their relative distance vs. speed of nucleus (E and F) were measured. The position and the speed correlation graphs indicate a strong coupling of the nucleus and the centrosome in both cell lines. Solid lines in A–D plots correspond to the correlation coefficient of 1 (red lines), while the dashed lines mark the linear fits. There is a weak negative correlation between the speed of nucleus and nucleus-centrosome distance (E and F)^*^. Here the red dashed lines separate the coordinate quadrants (for easier visualization) and the dashed blue line is the fitted linear regression. The grey area indicates the average half-length of the elongated nucleus (schematic representation below the plots) under these conditions (8.5 μm). Pearson's correlation coefficients are included in the plots. N = 32 (C6 cells) and N = 56 (U87 cells). ^*^ Note that these are center-to-center distances.

In order to gain a quantitative understanding of nucleus-centrosome connections, the positions and speeds of nuclei and centrosomes were calculated from the tracking data obtained previously (Figure 5 A–D). Both positions (Pearson's correlation coefficient: 0.99 for C6 cells, 0.89 for U87 cells, respectively) and speeds of nuclei and centrosomes (Pearson's correlation coefficient: 0.95 for C6 cells, 0.90 for U87 cells) were strongly correlated. A weak negative correlation between the speeds of the nuclei and nucleus-centrosome distances was also detected (Figure 5 E and F, Pearson's correlation coefficient: −0.29 for C6 cells, −0.25 for U87 cells). This negative correlation means that faster movements of the nuclei are linked to larger nucleus-centrosome distances. These distances themselves were clearly cell-line specific. In C6 cells, the average nucleus-centrosome distance was smaller than the typical half-length of the elongated nuclei (8.5 μm), indicating that the 2D-position of the centrosomes often overlapped with the ellipsoid of the nuclei (areas shaded in grey and drawings on Figure 5 E and F). During oscillations, the centrosome was thus always in close proximity of the nucleus. In contrast, in U87 cells distances of up to 40 μm were measured between the centroid of the nucleus and the centroid of the centrosome (Figure 5 F). Interestingly, the centrosome seemed to move in connection with the ER (Movie S4) in this cell line. Overall, in relation to the mean nucleus-centrosome distances we have observed significant fluctuations, especially in U87 cells. This indicates a dynamic linkage between these organelles.

**Figure 6:**
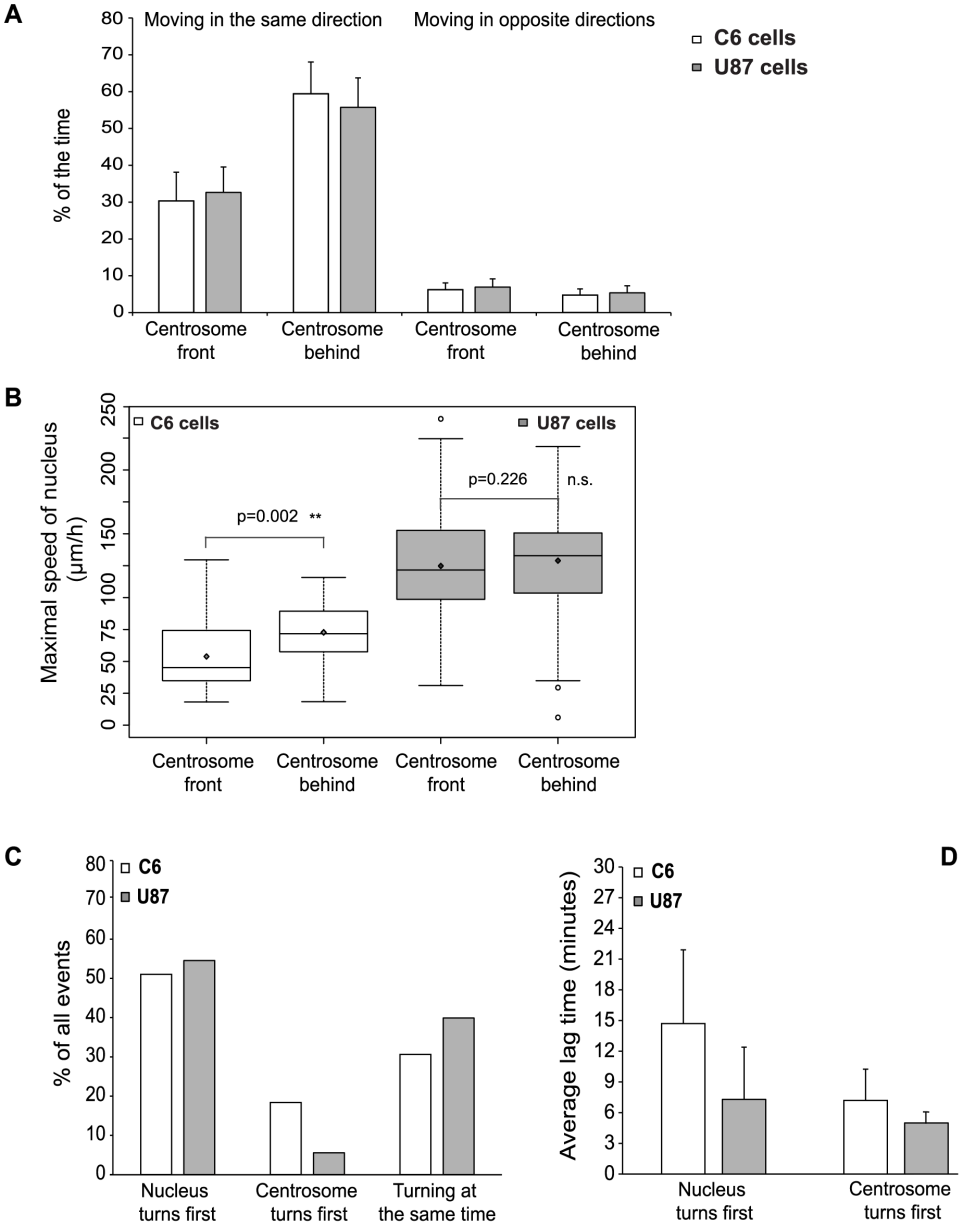
The centrosome rather follows than leads the nucleus during nuclear oscillations. (A) Centrosome positions relative to the center of the nucleus along the movement axis/vector determined by the nucleus. The centrosome is mostly behind. (B) Maximal speed of the nucleus as a function of the relative position of the centrosome. The nucleus moves faster when the centrosome is behind. Statistical significance was calculated using paired Wilcoxon signed rank test. Significance codes: ^*^: p<0.05, ^**^: p<0.01, (C) Frequency of the various scenarios of directional changes (nucleus turns first/centrosome turns first/turning at the same time). The turning is rather initiated by the nucleus, and followed by the centrosome (D) Lag time (in minutes) necessary for the nucleus or the centrosome to follow the other organelle for the “turning scenarios” presented on (C). The centrosome follows the nucleus more slowly than *vice versa*.

Although the measured correlations show that nuclear and centrosome movements are coupled, they do not reveal whether the centrosome is the driver of nuclear movements or a passenger of nuclear positioning. Therefore, we examined the relative position of centrosome centroids with respect to the direction of nuclear movement. The nucleus and the centrosome were predominantly moving in the same direction in both cell lines with the centrosome frequently positioned behind the nucleus (Figure 6 A, 66±9% of the time in C6 cells and 63±6% of the time in U87 cells). Despite these observations, it seems that the relative position of the centrosome has no major effect on the direction of the nuclear movements. On the other hand, the nucleus moved clearly faster, when the centrosome was located behind the nuclear centroid (Figure 6 B). This difference was more pronounced in C6 cells (median ± IQR of maximal nuclear speed: centrosome in front: 45±38 μm/h vs. centrosome behind: 72±31 μm/h) than in U87 cells (centrosome in front: 121±53 μm/h vs. centrosome behind: 133±47 μm/h).

To clarify the importance of elongated cell morphology in centrosome positioning, we studied nucleus and centrosome movements also within cells migrating on homogenous fibronectin surface. Overall we found that there was a small, but significant bias in centrosome positioning, with a preference for localization of the centrosome behind the nucleus (Figure S8: 54±9% of all frames in C6 cells, 58±10% of all frames in U87 cells). This slight bias compared to cells grown on patterns indicate that mechanical constrains caused by the patterning may play a role in centrosome positioning.

To investigate the chronological relationship between nuclear and centrosomal movements, we analyzed the temporal shifts between their periodic peaks. The nucleus most frequently reached the peak of its periodic curve before the centrosome (Figure 6 C, 51% vs. 18% of total events in C6 cells and 55% vs. 6% of total events in U87 cells). This indicates that the nucleus mostly changed its direction before centrosome reorientation. Upon reversal, the centrosome lags behind the nucleus longer than the nucleus behind the centrosome (Figure 6 D, estimated lag time of the centrosome is 15±14 min in C6 cells, and 7±5 min in U87 cells, whereas estimated lag time of the nucleus is 7±3 min in C6 cells and 5 min in U87 cells, respectively). Analysis of the cross-correlation data between the centrosome and the nuclear positions in individual cells revealed that while in C6 cells the centrosome frequently lags behind the nucleus, they also often move synchronously (Figure S9, lag = 0 (below our detection threshold)). In U87 cells, we obtained similar results. In summary, these results indicate that centrosome positioning itself may not have a major role in nuclear/cellular oscillations induced by topographical cues.

## Discussion

The mechanical processes underlying nuclear movements of mammalian cells are far from being understood. In our studies, we used a micro-patterning based assay to induce oscillatory nuclear movements in glioma cell lines with the aim of revealing molecular and mechanical details of nuclear migration and positioning. Similar micro-fabrication techniques [56], [59] have been applied in order to mimic ‘naturally existing’ morphological cues of the tissue microenvironment in cell culture systems.

In accordance with previous data [43], perturbing microtubule dynamics interfered with nuclear movements in C6 cells. Further inhibitor experiments indicated that intact actin and microtubule cytoskeletons are necessary to maintain oscillation within both cell lines, although U87 cells were more sensitive to actin and myosin inhibition, than C6 cells (Figure 2 A and C). Considering that actin is not only a force-generator but also involved in maintenance of nuclear shape and nuclear orientation in mammalian cells, it is likely that the latter role also contributes to the observed effects (slower nuclear migration speed, large fraction of non-moving cells) [24], [60], [61]. Remarkably, the cell extensions of blebbistatin-treated U87 cells became stationary, while some nuclei still displayed oscillatory movements (Figure S6). Since the movement of the nucleus was strongly coupled to cell migration in this cell line, it is likely that inhibition of actomyosin contractility decoupled cell migration from nuclear movements in U87 cells. In contrast, blebbistatin treatment stimulated oscillatory movements in C6 cells (Figure 2 A and B). Increase in nuclear motility and in proportion of bipolar C6 cells upon blebbistatin treatment were also shown by a recent study [45]. Separate tracking of the nucleus and the cell centroid revealed that myosin inhibition also increased cell speed, not only the speed of the nucleus (Figure 3). Therefore, it is rational to argue that such enhanced nuclear movements are caused to some extent by the increased cell motility.

### Nuclear movements in C6 cells require dynein activity

As the role of dynein/dynactin complex in nuclear positioning is well established from studies performed in several model systems [62]–[65], dynein was a valid candidate to support oscillatory movements of nuclei in our system. In our experiments, dynein inhibition alone was enough to block nuclear movements of C6 cells, even in the presence of active myosin (Figure 2 A and B, Figure 3 A). Moreover, treatment with blebbistatin in combination with EHNA only weakly reverted the effect of dynein inhibition (Figure 3 A and B, Figure S5), implying that dynein is a dominant force-generator establishing nuclear movements in C6 cells. Notably, cell migration on homogenous fibronectin surfaces was unaffected by EHNA in any of the cell lines (Figure S5), suggesting that while dynein activity is required for facilitating 1D nuclear movements, it has a less pronounced role in 1D cellular movements and 2D cell migration. The contextual dependence of dynein and myosin inhibition was also proposed by a recent study, which showed that dynein inhibition had no effect on migration of cells on rigid surfaces but stopped movement of cells plated on soft gels, regardless of the presence of myosin activity [66].

### Centrosome position follows nuclear movements

The movements of the centrosome were strongly coupled to nuclear positioning in oscillating C6 and U87 cells (Figure 5 A–D, Figure S9). However, the fluctuations of nucleus-centrosome distance indicated that the connection between nucleus and centrosome must be dynamic and mechanically flexible (Figure 5 E and F). A possible explanation for this observation is that the nucleus-centrosome connection is not direct, but it involves dynamic microtubules of variable length. In both cell lines, the centrosome was most frequently found behind the nucleus during oscillations, consistent with previous results [43], [45]. While the oscillatory motion itself was not significantly perturbed when the centrosome was positioned in front of the nucleus, a position behind the nucleus resulted in faster nuclear movements in C6 cells (Figure 6 B). Pushing forces generated by microtubule polymerization from the centrosome may also contribute to nuclear movements as suggested previously [43]. Alternatively, tight association of the centrosome with other membranous organelles like the Golgi or the ER [15], [67] may create a physical barrier for nuclear movements [68] and consequently the nucleus can move faster in the direction of a “less viscous” environment, i.e. with the centrosome behind.

Our observations of the temporal order of nucleus-centrosome reorientation in 1D cells (i.e. the nucleus turns back before the centrosome, Figure 6 and Figure S9) are clearly different from previous studies about this phenomenon. During nucleokinesis, the movement of the centrosome precedes nuclear displacements; therefore, the centrosome -via nucleation of a “perinuclear cage” consisting of microtubules- is suggested to lead the nucleus [17], [69]. In wound-edge fibroblasts, the centrosome is stationary, while the nucleus moves away from the leading edge of the cells [41]. In another study, using cells plated on micro-patterns, centrosome movement towards the leading edge preceded the displacement of the nucleus during cell polarization [70]. These controversial observations indicate that centrosome position relative to the nucleus generally is not a key predictor of nuclear movements, but is rather a system/experiment dependent factor. This interpretation is strengthened by the fact that the directional changes of the nucleus most frequently preceded centrosome reorientation in both cell lines (Figure 6 C), suggesting that the main force driving the nuclear movements is applied directly on the nucleus, and is not transmitted via the centrosome.

### Behavior differences between the two cell lines can be explained by cellular tension differences

The two investigated cell lines showed remarkable similarities but also differences in their behavior and response to drug treatments. We suggest that the various types of oscillations (nuclear vs. whole cell movements) are present in both investigated cell lines but their relative importance are primarily determined by the cytoskeletal/cellular tension. We are referring to this cytoskeletal/cellular tension as a general mechanical tension continuously present in the cells. Cytoskeletal tension is mainly generated by the actomyosin network, and resisted by external connections (to extracellular matrix or to neighboring cells) and internal load-bearing structures (e.g. microtubules) [71].

**Figure 7:**
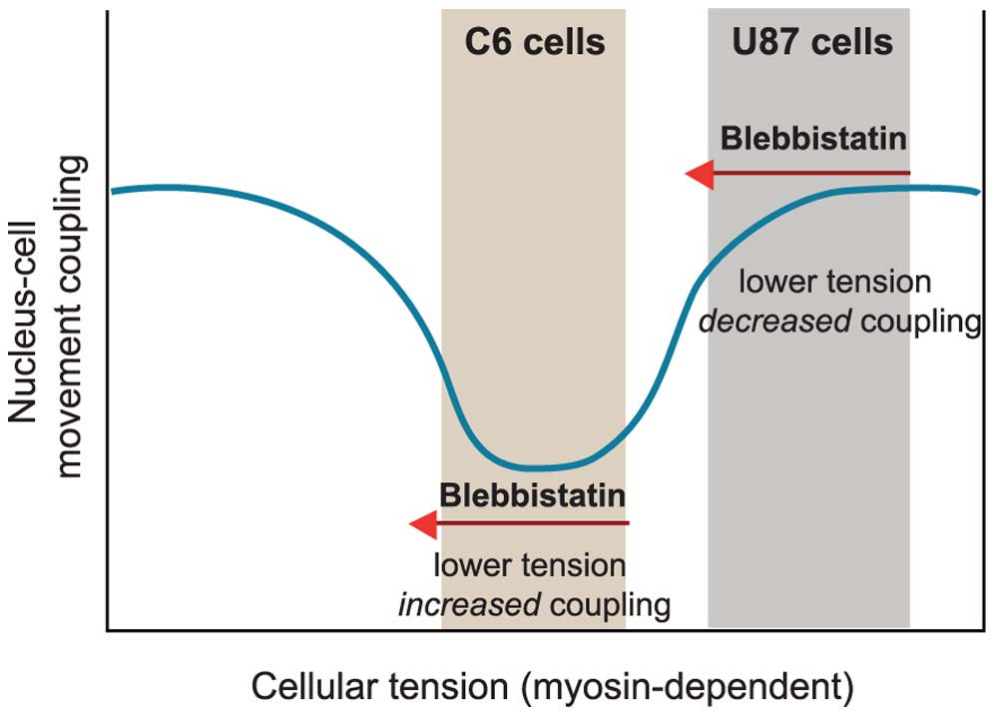
Relationship of nucleus-cell movement coupling and cellular tension. The different nuclear migration phenotypes observed in the C6 and U87 cells can be explained by assuming a bimodal relationship between cytoskeletal tension (determined mainly by the contractility of the actomyosin network) and the mechanical coupling of nuclear and cellular movements. Relatively high coupling can be achieved at both low and high level of cytoskeletal tension, whereas the medium tension values correspond to weaker nucleus-cell movement coupling. Based on our results, we propose that C6 cells are normally in the range of medium tension, whereas U87 cells populate the high-tension range. Reducing the network contractility (and hence the cytoskeletal tension) by blebbistatin shifts U87 cells towards the tension range usually occupied by C6 cells, and results in a nuclear migration phenotype similar to the C6 cells. The same inhibition moves C6 cells into the range of lower tension, and produces a phenotype with increased lamellipodia activity linked to increased cellular migration and a stronger coupling between the cell and nucleus movements.

Nuclear oscillation is dynein dependent (see also the next section) and can be repressed by high cellular tension. This type of movement is predominant in geometrically confined C6 cells, and to a lesser extent in U87 cells. In U87 cells the blebbistatin treatment may trigger the autonomous nuclear oscillations via its inhibitory effect on the whole cell oscillations. If we assume a bimodal relationship between cytoskeletal tension and the mechanical coupling of nuclear and cellular movements, then high degree of coupling could be achieved at both low- and high values of cytoskeletal tension, whereas medium levels of tension would correspond to a weaker nucleus-cell movement coupling (Figure 7). Following this argumentation/model, as C6 cells show a weak coupling of cell-nucleus movements in their natural/control state (Figure S3), we place them in the medium tension regime in this model. U87 cells on the other hand showed strong coupling between the cellular and nuclear movements (Figure S3), which sets them in the higher tension domain (Figure 7). Reducing non-muscle myosin II activity, thus cytoskeletal tension by blebbistatin [72], [73] shifts U87 cells towards the tension range usually occupied by C6 cells, resulting in a weaker coupling and in a “true” nuclear migration phenotype very similar to C6 cells (Figure S6 and S7). The same drug treatment on the other hand positions C6 cells into the range of low cytoskeletal tension where the coupling between the movements of the nucleus and cell is again stronger (Figure S7). In agreement with our rationale, in bipolar C6 cells, relatively low traction forces were reported [45]. Moreover, substrate rigidity/cellular tension- dependent effects of blebbistatin treatment have also been shown in glioma cells and several other cell lines [74], [75]. Obviously, other factors than myosin – cell shape, cell-matrix adhesions, and cell spreading area – may contribute to tension [76]–[78] and hence, further experiments are needed to confirm the suggested model.

### Model of nuclear migration

**Figure 8:**
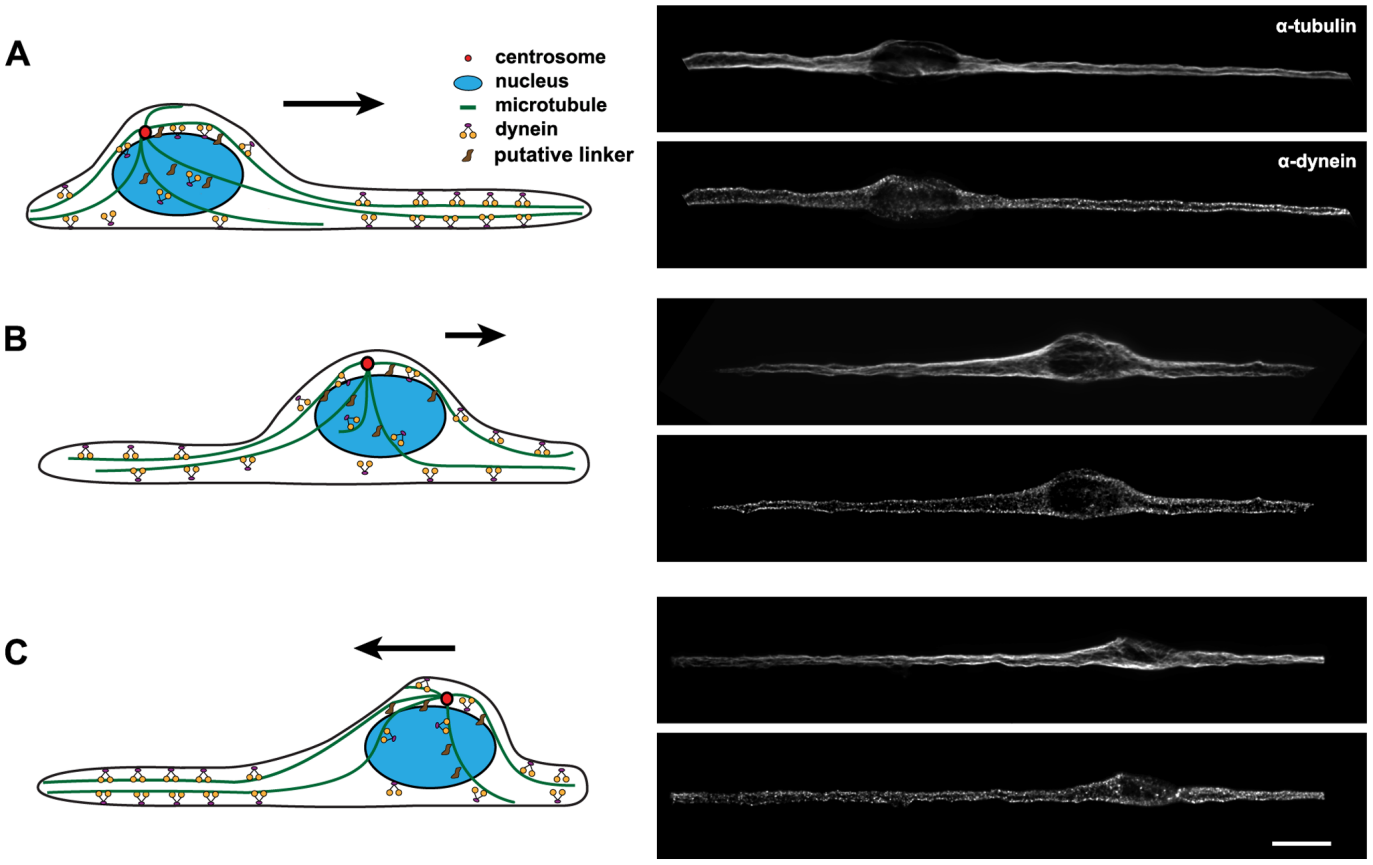
Model of nuclear oscillations. The main driving force of nuclear movements in C6 cells is generated by cortically anchored cytoplasmic dynein, and transmitted via microtubules to the nucleus. Microtubule-nucleus connections may be mediated via the collaboration of microtubule motors and/or linker proteins. Labels illustrate distinct phases of the oscillatory movement. On the right side of the figure, a single z-section of representative C6 cells with tubulin (upper panels) and dynein (lower panels) is shown (scale bar =  15 μm). (A) The number of motors (exerted force) is proportional to filament length, and determines the movement direction. (B) As the nucleus approaches the cell edge, the leading microtubules shorten, whereas the trailing ones become longer so more motors can attach to them. (C) Now the trailing microtubules are able to pull stronger on the nucleus than the leading ones –so the nucleus reverses its direction. Arrows indicate the direction of nuclear movement.

In the case of C6 and myosin II-inhibited U87 cells, we clearly observed the existence of “true” nuclear migration, i.e. nuclear movement relative to the cell extensions, independently of the rest of the cell. Below, we speculate about a mechanical model in which the driving forces governing nuclear oscillations are generated by cytoplasmic dynein and microtubule dynamics, but are not directly dependent on the centrosome. Our explanation mainly follows the argumentation of the model presented for the meiotic nuclear oscillations in *Schizosaccharomyces pombe* [4], [79], [80]. We assume that the major forces acting on the nucleus are determined by the number of contributing dynein complexes and microtubule dynamics (Figure 8). To exert force on the nucleus, dynein must be connected to both the cell cortex and - via microtubules - to the nucleus (Figure 8, Movie S6). Nucleus-microtubule connections are likely to be mediated by molecular motors associated with linker proteins or the nuclear pore complexes [21], [34], [47], [81], [82]. Our model further assumes that cortically anchored dynein motors generate the pulling forces acting on the nucleus (Figure 8 A). Although dynein-cell cortex interactions are relatively unexplored within interphase mammalian cells, the existence of such connections has already been shown [83]–[87]. Immunostained images displaying dynein and microtubule localization during the various stages of nuclear migration (Figure 8, Movie S6) also suggest the plausibility of our model.

Microtubule length and number may be important factors governing nuclear oscillations in C6 cells. Live cell imaging of EB3-expressing C6 cells confirmed an asymmetry in microtubule growth rate (Figure S10, Movie S5), correlating with the direction of nuclear movements. While the nucleus moves in the cell, leading microtubules shorten, whereas trailing microtubules grow, thus more motors may in principle attach on the trailing side, and the force opposing the actual direction of movement may increase (Figure 8 B). As the nucleus approaches the cell edge, the pulling forces exerted by the trailing microtubules would eventually exceed the leading ones and cause the nucleus to reverse its direction (Figure 8 C). The possible balance of forces in the center of the cell could be overcome by the load-dependent redistribution of dynein complexes [80], [88], and changes in microtubule dynamics and/or numbers during oscillations.

At the current state of our research we cannot determine which of the above listed two effects are more important, and since they are not mutually exclusive, it seems most likely that a combination of them drives nuclear oscillations in C6 cells. In contrast to C6 cells, nuclear oscillations of U87 cells are strongly coupled to overall cell motility; hence they cannot solely be explained with this model. Nevertheless, we propose that the same dynein-dependent forces are exerted on the nucleus in this cell line, but may be masked by additional contributions of actomyosin contraction. When blocking the actomyosin network activity by blebbistatin treatment, one can observe pure nuclear migration, and it can be mechanically explained using the model presented for the C6 cells.

Densely packed microtubules in bipolar C6 and U87 cells cannot be sufficiently resolved to understand their exact organization by conventional fluorescence microscopy. In migrating granule cells, microtubules connecting the nucleus to the leading edge were shown to be centrosome-independent, and the cage-like microtubule structure surrounding the nucleus did not converge at the centrosome [40]. Release of microtubules from the centrosome and their stabilization elsewhere in the cell has also been shown in mammalian cells [89]. To unravel the role of the microtubule cytoskeleton in micro-pattern induced nuclear oscillations, it will therefore be crucial to elucidate microtubule organization in U87 and C6 cells at a high resolution. Correlative light and electron microscopy or super-resolution light microscopy may provide detailed insights in the future.

## Supporting Information

Figure S1 **Actin and tubulin cytoskeleton rearrangements induced by geometrical constrains in C6 cells.** C6 cells were seeded on homogenous fibronectin (A and B) or micro-patterned fibronectin surfaces (C and D). Actin and microtubules were labeled with coumarin-phalloidin (A and C) and alpha-tubulin antibody (B and D), respectively. Inverted monochromatic lookup tables of C6 cells presented in Figure 1 reveal more details of F-actin and microtubule structures under both conditions. Highlighted regions of C and D show the alignment of actin and microtubule filaments (arrows) along to the fibronectin pattern. Scale bars:10 μm.
(TIF)

Figure S2 **Categorization of nuclear movements in U87 cells.** Based on the coordinates of nuclei projected to the movement axis (i.e. along the pattern) and visual inspection of their corresponding trajectories, we have established the following categories: (A) Oscillatory movement: nuclei display a periodic movement along the pattern in at least 80% of the measured time. (B) Irregular movement: nuclei move without recurrent periodicity. (C) No movement: nuclei show no significant positional change over most of the time. This means that the cumulative nuclear displacement within 14 hours was below 200 μm for C6 cells, or below 300 μm in the case of U87 cells.
(TIF)

Figure S3 **Coupling between nuclear migration and cellular movements.** Cell extensions and nuclei of C6 and U87 cells seeded on patterns were manually tracked (n = 15). Representative example of an oscillating C6 (A) and U87 cell (B). Top panels: Positions of the cell center, the nucleus and the cell edges projected along the pattern over time. Middle panels: Relative position of the nucleus within the cell, normalized to the cell edges^*^. Allows visualizing the nuclear movements inside the cell. Lower panels: Related cross-correlation plots indicate no coupling between the movement of the nucleus and the cell centroid in C6 cells, and a strong correlation between their movements in U87 cells. Red vertical lines mark the lag at 0, red dashed lines indicate 95% confidence intervals. ^*^ Cell edges are defined at the start of tracking process, thus the “leading” or “trailing” edge terms are arbitrary.
(TIF)

Figure S4 **Microtubule and dynein inhibitors perturb nuclear oscillations in C6 cells.** C6 cells were plated on fibronectin patterns and treated either with solvent control (DMSO) or with cytoskeletal inhibitors during overnight imaging experiments. Representative kymographs (each consists of 100 frames) demonstrate the response of micro-patterned C6 cells to the various treatments. Time interval between two consecutive frames was 5 minutes. Scale bar: 20 μm.
(TIF)

Figure S5 **Distinct effects of myosin and dynein inhibition in C6 and U87 cells.** C6 (left) and U87 cells (right) were treated with 10 μM blebbistatin, 0.5 mM EHNA, or the combination of these drugs. Top row: proportion of cells in the different motility subgroups in 1D (cells seeded on the patterns). Middle row: average speed of the total cell population in 1D. Bottom row: average cell migration speed of C6 (left) and U87 (right) cells moving on 2D (homogenous fibronectin coating) surfaces. On the box plots, mean values are marked by diamonds, whereas empty circles represent outliers. Statistical analysis was performed using Kruskal-Wallis test on data of 2 independent experiments. Error bars indicate SE.
(TIF)

Figure S6 **Inhibition of non-muscle myosin II induces nuclear migration in U87 cells.** Kymographs of a representative solvent control (DMSO) and blebbistatin treated U87 cell. Upon non-muscle myosin II inhibition the nucleus oscillates slowly within the cell, but the cell edges remain stationary. Scale bar: 20 μm.
(TIF)

Figure S7 **Effects of myosin and dynein inhibition on nucleus-cell movement coupling.** Positions of nucleus and cell extensions over time in representative oscillating C6 (A) and U87 cells (B) subjected to various drug treatments. Note that myosin inhibition increases the range of nuclear oscillations in both cell lines. (C) Locations of the maximum cross-covariance values (mean ± SE) and the corresponding lags (mean ± SE) are plotted upon the different treatments in C6 and U87 cells. While in C6 cells, blebbistatin slightly increases nucleus-cell cross-correlations, and decreases the lag times; it lowers the correlation of nucleus-cell movements in U87 cells. Red lines crossing the plot indicate the control values. At least 10 cells per treatment from 3 independent experiments were analyzed.
(TIF)

Figure S8 **The centrosome is frequently behind the nucleus in cells migrating on patterned and non-patterned fibronectin surfaces.** Centrosome (red marked lines) and nucleus trajectories (black marked lines) of representative C6 (A) and U87 cells (B) moving on homogenous fibronectin-coated surfaces (2D). (C) Centrosome positioning relative to the nucleus and the direction of the cell migration in C6 and U87 cells on patterned vs. non-patterned fibronectin surfaces. Note that the centrosome is most frequently localized behind the nucleus both in geometrically constrained (1D) and freely migrating (2D) cells.
(TIF)

Figure S9 **Confirmation of centrosome lagging by centrosome-nucleus positional cross-correlation analysis.** Nucleus and centrosome positions of representative C6 and U87 cells (A) and their corresponding positional cross-correlation plots (B) illustrate the correlated movements of the nucleus and the centrosome in C6 and U87 cells. (C) Cross-correlation lags indicate that the centrosome either moves together or lags behind the nucleus in both cell lines.
(TIF)

Figure S10 **Microtubule dynamics of C6 cells revealed by YFP-EB3.** C6 cells were transiently transfected with YFP-EB3, seeded on fibronectin patterns and imaged with a spinning disc microscope (speed: 400 ms/frame). To estimate the direction of nuclear movements, short (2 minutes) phase contrast time-lapse series of the selected cells were taken preceding YFP-EB3 imaging. (A) Inverted LUT (lookup table) image shows a C6 cell expressing the YFP-EB3 marker. This single image represents the maximum intensity projection of 50 time points. Scale bar: 10 μm. (B) Kymograph of YFP-EB3 along the dashed line marked in (A), indicating that there are more microtubules growing in front of the nucleus than behind. Scale bar: 5 μm. (C) Method for calculating microtubule growth rate. Speed (rate of microtubule polymerization)  =  travelled EB3 distance/time. (D) Histograms of microtubule growth in the direction of nuclear movement (front of the nucleus) and opposite of that measured within the same cells (n = 50 slopes of each direction, data obtained from 8 cells). Statistical analysis was performed using paired Wilcoxon test. Black lines overlaid on histograms indicate probability densities.
(TIF)

Table S1 **Influence of cytoskeletal and motor protein inhibitors on oscillating cells.**
(DOCX)

Movie S1 **Geometrical constrains modify cell shape and motility of C6 cells.** C6 rat glioma cells migrating on homogeneous (left) or patterned fibronectin-fibrinogen coated surface (right). Playback speed: 9 frames per second.
(MOV)

Movie S2 **Geometrical constrains modify cell shape and motility of U87 cells.** U87 human glioma cells migrating on homogeneous (left) or patterned fibronectin-fibrinogen coated surface (right). Playback speed: 9 frames per second.
(MOV)

Movie S3 **Nucleus-centrosome coupling in C6 cells during nuclear oscillation.** Time-lapse movie of a GFP-centrin-2 (green) expressing C6 cell moving along a 200/5 μm fibronectin rectangle. The nucleus was labeled with Hoechst 3342 prior to imaging. Playback speed: 9 frames per second.
(MOV)

Movie S4 **Nucleus-centrosome coupling in U87 cells during nuclear oscillation.** U87 cells expressing GFP-centrin 2 are oscillating on a fibronectin pattern. The track of a centrosome (red dot) is merged with the phase contrast picture of the corresponding cell. Note that the nucleus does not rotate during oscillations. Playback speed: 7 frames per second.
(MOV)

Movie S5 **Microtubule polymerization in bipolar C6 cells.** C6 cells expressing a microtubule plus-end marker (YFP-EB3) were seeded on fibronectin patterns, and imaged with a spinning disc microscope (400 ms/frame). Note that the majority of microtubule plus ends are moving from the centrosome towards the cell periphery. Playback rate: 3 frames per second.
(MOV)

Movie S6 **3D dynein localization in C6 cells.** The animation displays a 360° rotation of a C6 cell (maximum intensity projection shown in 3D, Imaris) fixed after live cell imaging. Microtubules are shown in red, dynein is in green. The nucleus (blue) was moving towards the left prior to fixation. The first frames of the movie show the top view of the cell. Note that dynein is distributed along microtubules and around the nucleus.
(MOV)
