# Supplementary figures and images for: Nuclear Motility in Glioma Cells Reveals a Cell-Line Dependent Role of Various Cytoskeletal Components
Source: PLoS One. 2014 Apr 1;9(4):e93431. doi: 10.1371/journal.pone.0093431 (PMC3972233; doi:10.1371/journal.pone.0093431)

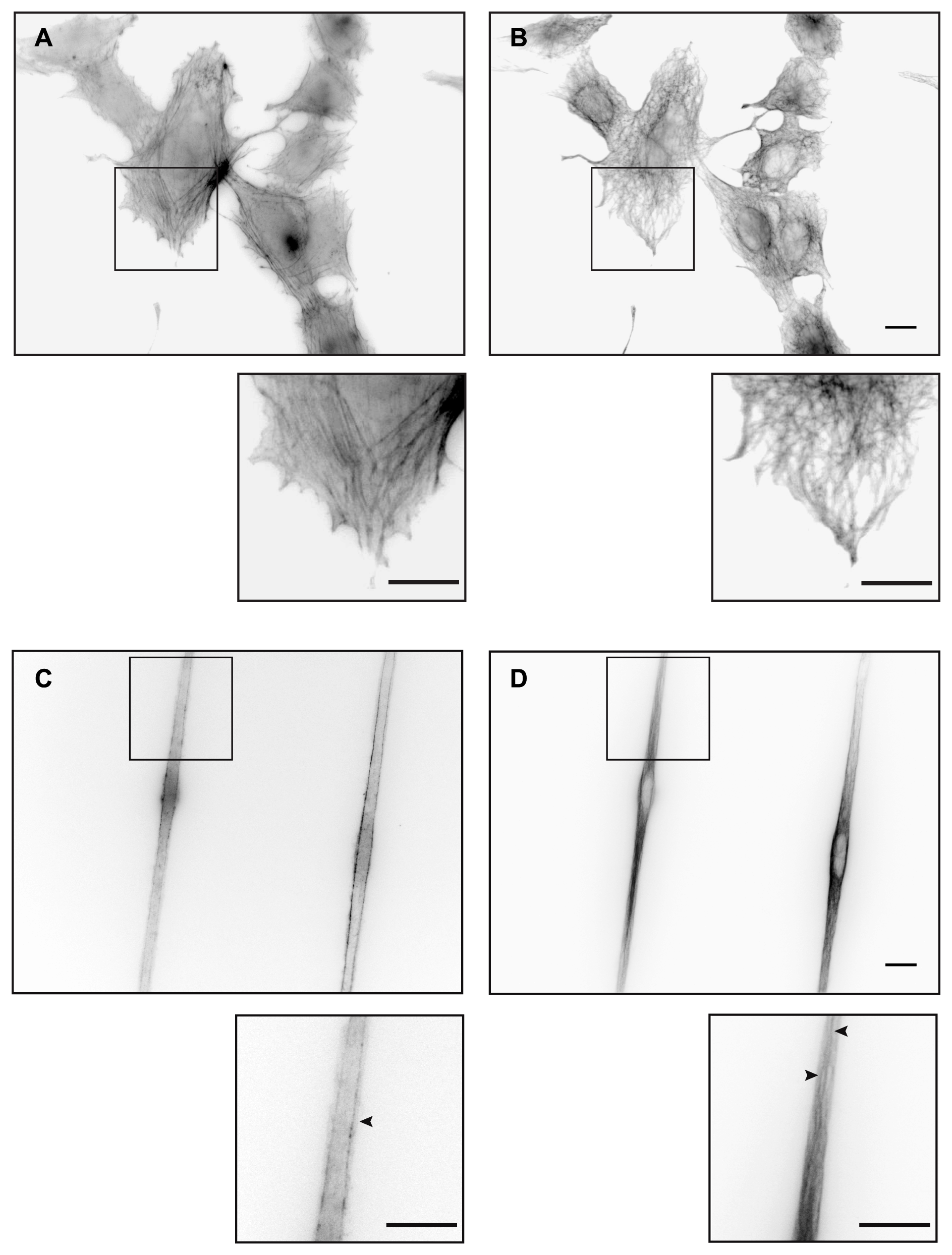

Supplement: Figure S1 — Actin and tubulin cytoskeleton rearrangements induced by geometrical constrains in C6 cells. C6 cells were seeded on homogenous fibronectin (A and B) or micro-patterned fibronectin surfaces (C and D). Actin and microtubules were labeled with coumarin-phalloidin (A and C) and alpha-tubulin antibody (B and D), respectively. Inverted monochromatic lookup tables of C6 cells presented in Figure 1 reveal more details of F-actin and microtubule structures under both conditions. Highlighted regions of C and D show the alignment of actin and microtubule filaments (arrows) along to the fibronectin pattern. Scale bars:10 μm. (TIF) [file pone.0093431.s001.tif]

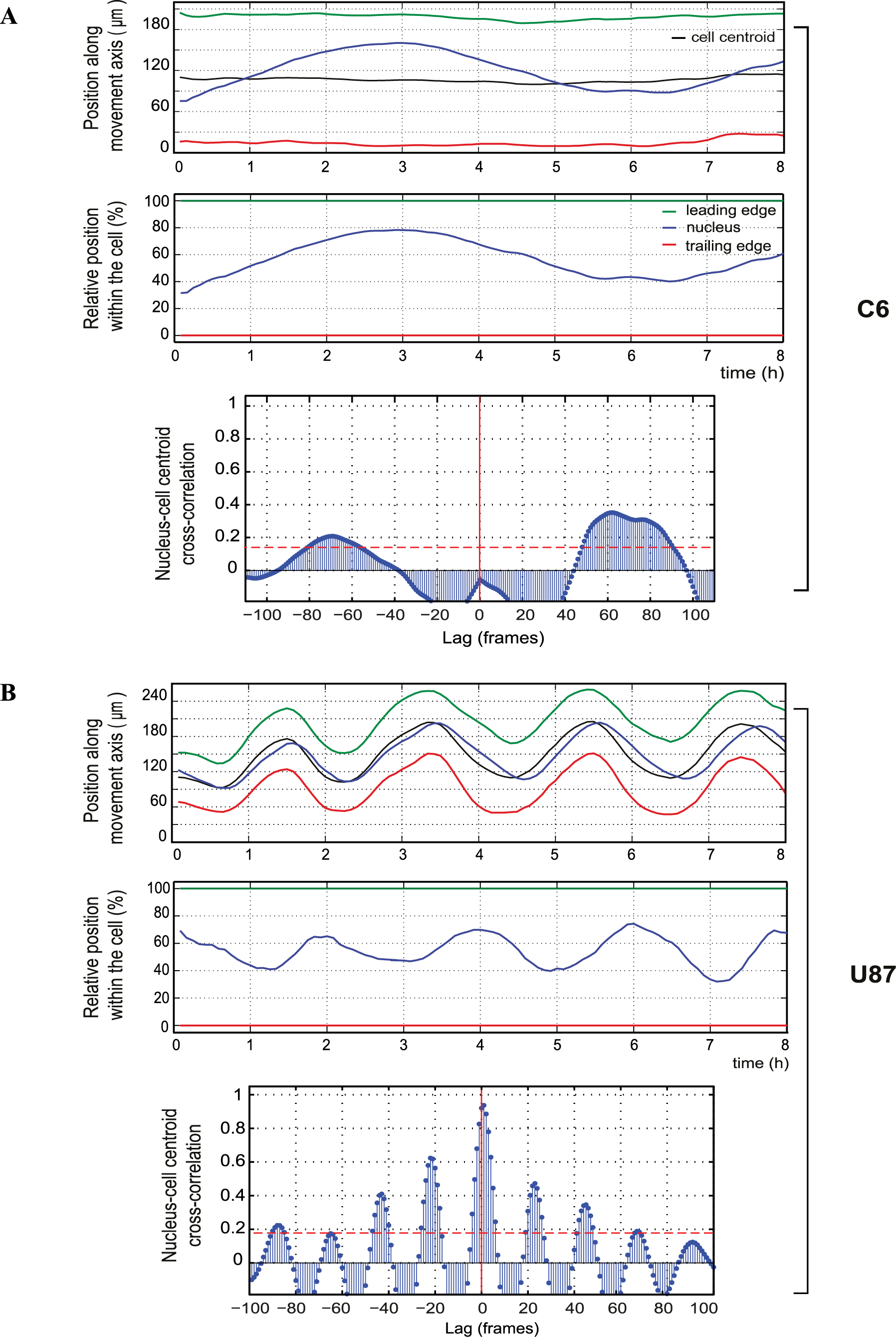

Supplement: Figure S2 — Categorization of nuclear movements in U87 cells. Based on the coordinates of nuclei projected to the movement axis (i.e. along the pattern) and visual inspection of their corresponding trajectories, we have established the following categories: (A) Oscillatory movement: nuclei display a periodic movement along the pattern in at least 80% of the measured time. (B) Irregular movement: nuclei move without recurrent periodicity. (C) No movement: nuclei show no significant positional change over most of the time. This means that the cumulative nuclear displacement within 14 hours was below 200 μm for C6 cells, or below 300 μm in the case of U87 cells. (TIF) [file pone.0093431.s002.tif]

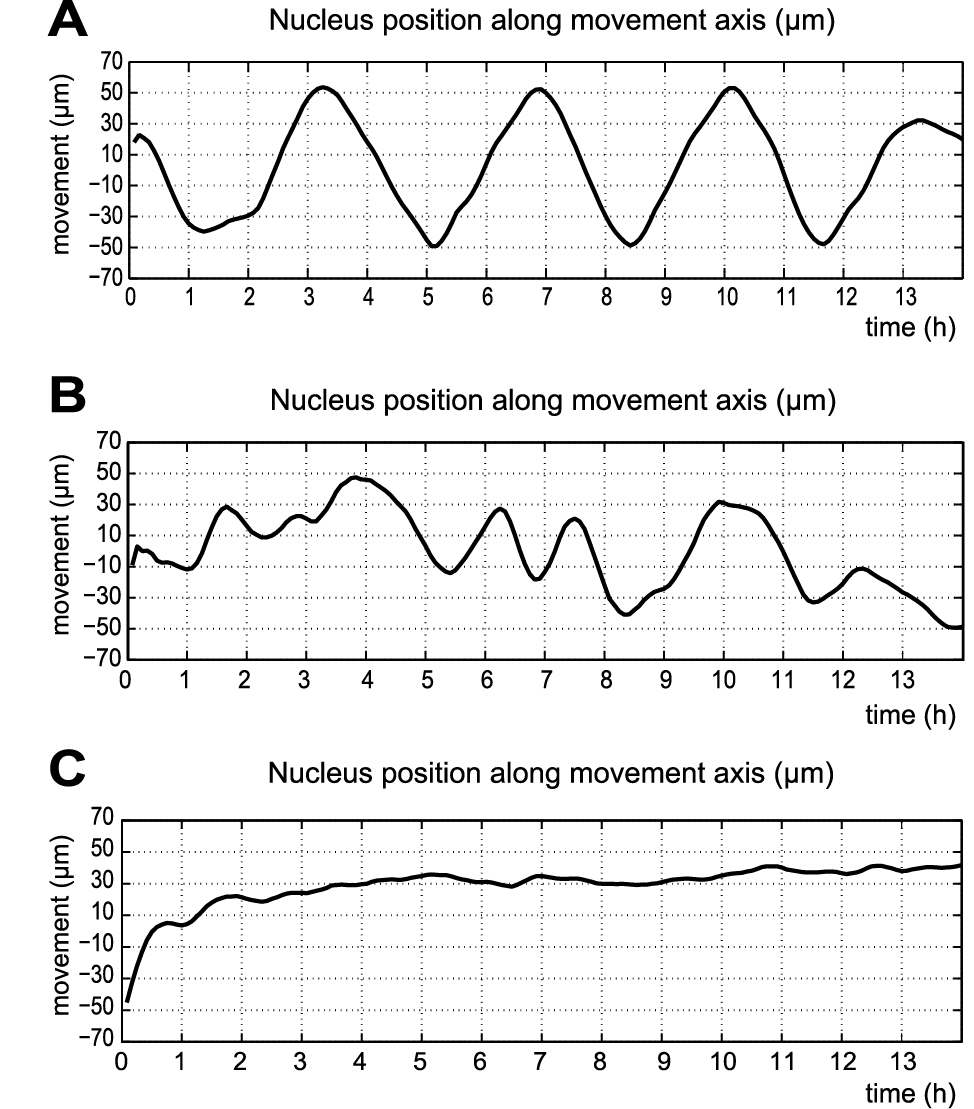

Supplement: Figure S3 — Coupling between nuclear migration and cellular movements. Cell extensions and nuclei of C6 and U87 cells seeded on patterns were manually tracked (n = 15). Representative example of an oscillating C6 (A) and U87 cell (B). Top panels: Positions of the cell center, the nucleus and the cell edges projected along the pattern over time. Middle panels: Relative position of the nucleus within the cell, normalized to the cell edges*. Allows visualizing the nuclear movements inside the cell. Lower panels: Related cross-correlation plots indicate no coupling between the movement of the nucleus and the cell centroid in C6 cells, and a strong correlation between their movements in U87 cells. Red vertical lines mark the lag at 0, red dashed lines indicate 95% confidence intervals. * Cell edges are defined at the start of tracking process, thus the “leading” or “trailing” edge terms are arbitrary. (TIF) [file pone.0093431.s003.tif]

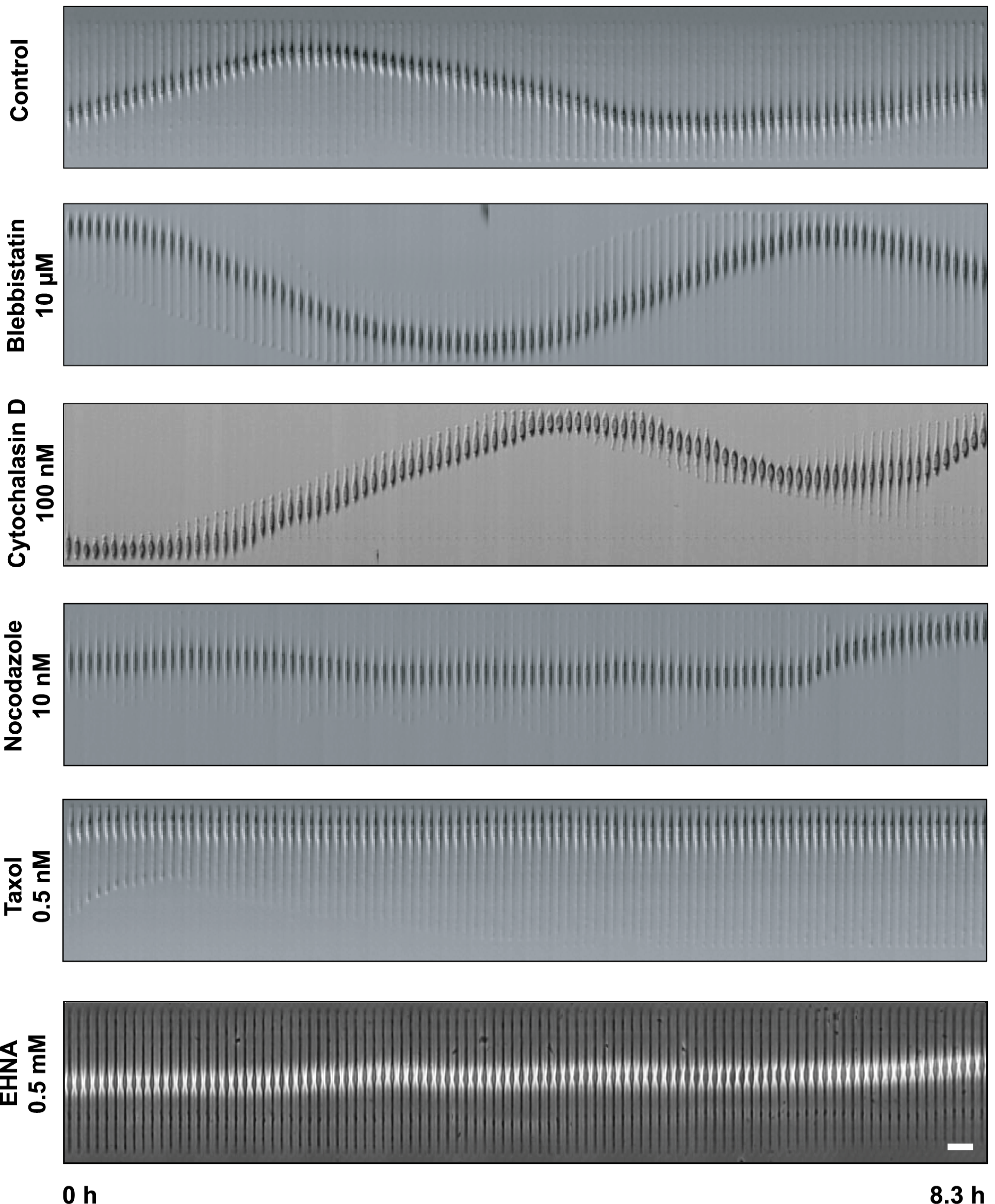

Supplement: Figure S4 — Microtubule and dynein inhibitors perturb nuclear oscillations in C6 cells. C6 cells were plated on fibronectin patterns and treated either with solvent control (DMSO) or with cytoskeletal inhibitors during overnight imaging experiments. Representative kymographs (each consists of 100 frames) demonstrate the response of micro-patterned C6 cells to the various treatments. Time interval between two consecutive frames was 5 minutes. Scale bar: 20 μm. (TIF) [file pone.0093431.s004.tif]

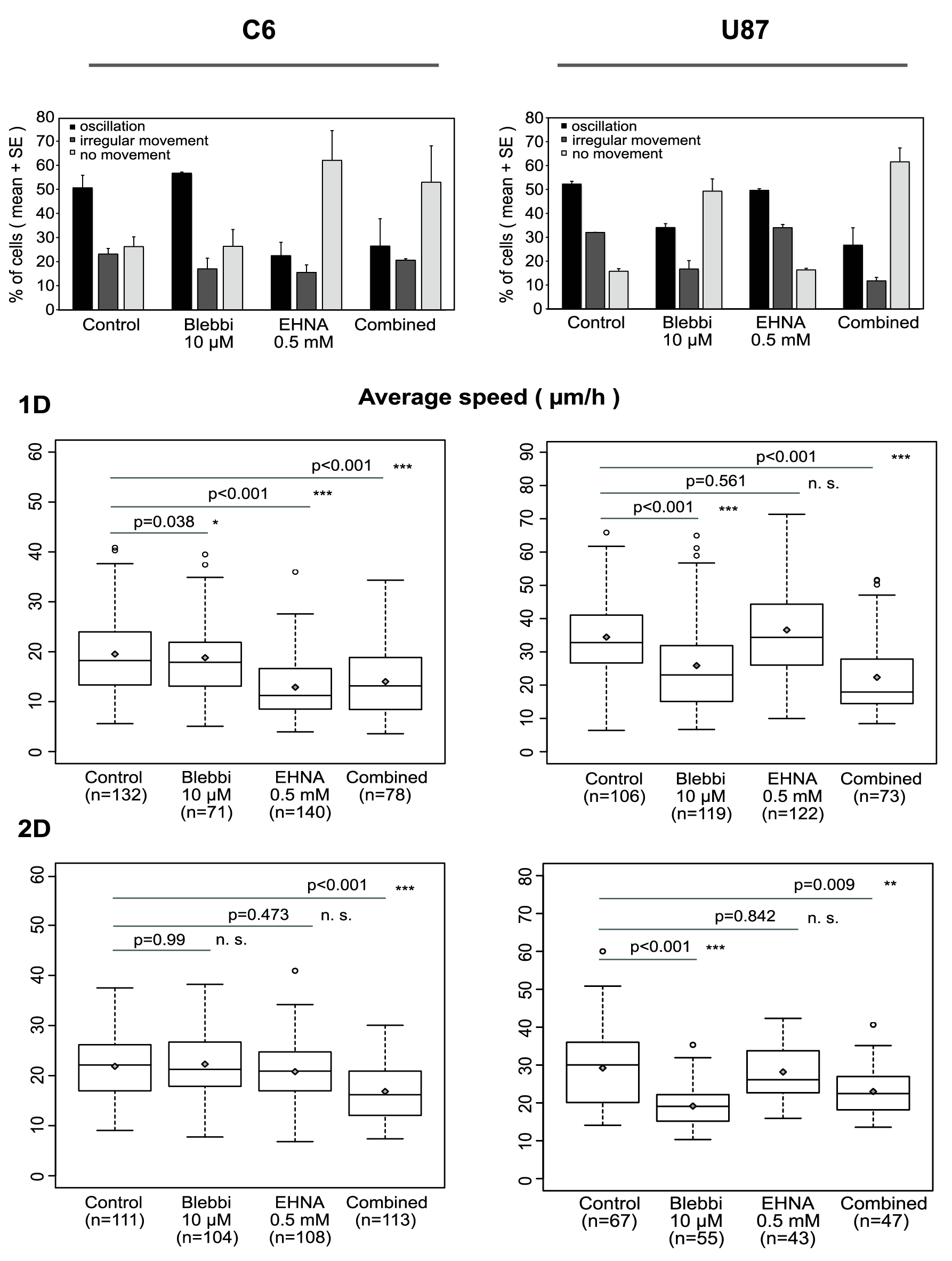

Supplement: Figure S5 — Distinct effects of myosin and dynein inhibition in C6 and U87 cells. C6 (left) and U87 cells (right) were treated with 10 μM blebbistatin, 0.5 mM EHNA, or the combination of these drugs. Top row: proportion of cells in the different motility subgroups in 1D (cells seeded on the patterns). Middle row: average speed of the total cell population in 1D. Bottom row: average cell migration speed of C6 (left) and U87 (right) cells moving on 2D (homogenous fibronectin coating) surfaces. On the box plots, mean values are marked by diamonds, whereas empty circles represent outliers. Statistical analysis was performed using Kruskal-Wallis test on data of 2 independent experiments. Error bars indicate SE. (TIF) [file pone.0093431.s005.tif]

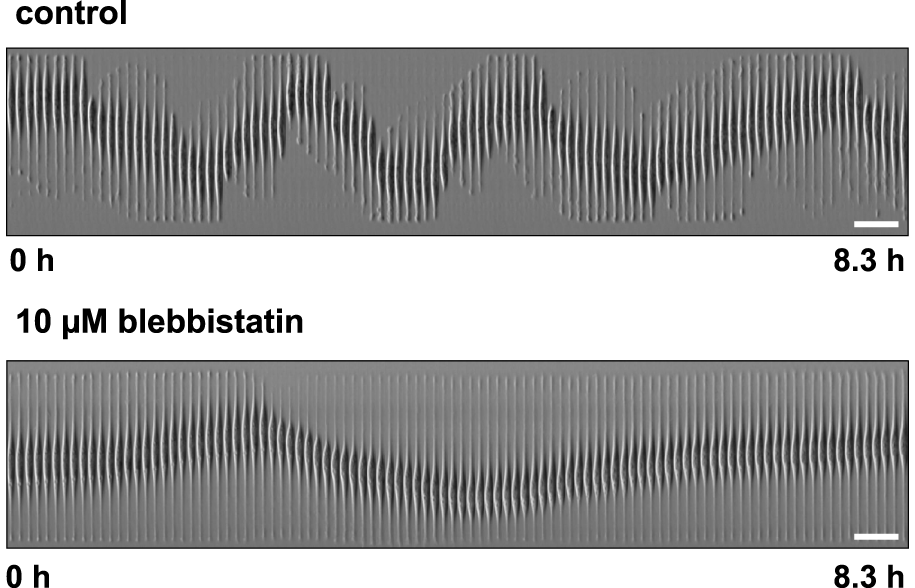

Supplement: Figure S6 — Inhibition of non-muscle myosin II induces nuclear migration in U87 cells. Kymographs of a representative solvent control (DMSO) and blebbistatin treated U87 cell. Upon non-muscle myosin II inhibition the nucleus oscillates slowly within the cell, but the cell edges remain stationary. Scale bar: 20 μm. (TIF) [file pone.0093431.s006.tif]

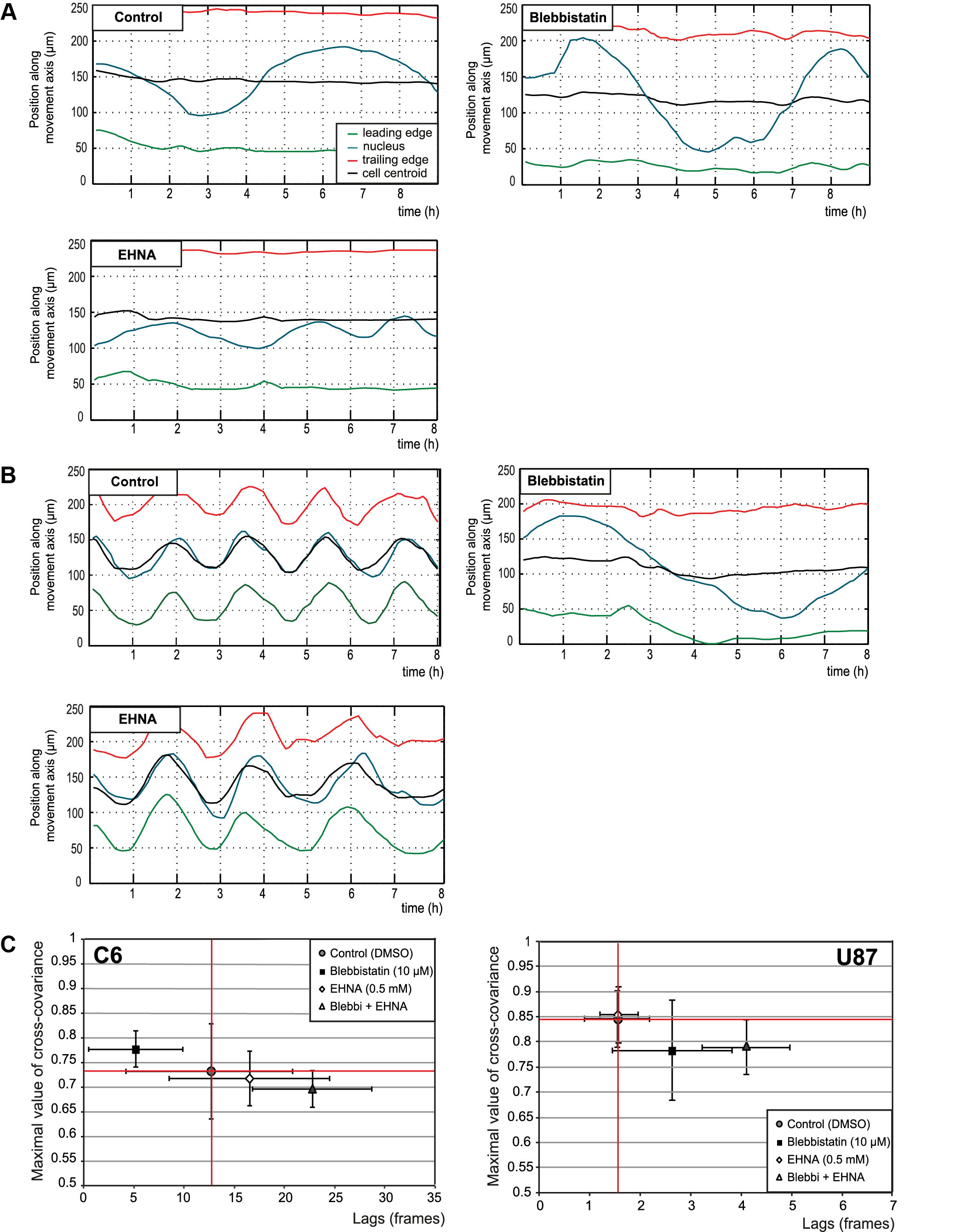

Supplement: Figure S7 — Effects of myosin and dynein inhibition on nucleus-cell movement coupling. Positions of nucleus and cell extensions over time in representative oscillating C6 (A) and U87 cells (B) subjected to various drug treatments. Note that myosin inhibition increases the range of nuclear oscillations in both cell lines. (C) Locations of the maximum cross-covariance values (mean ± SE) and the corresponding lags (mean ± SE) are plotted upon the different treatments in C6 and U87 cells. While in C6 cells, blebbistatin slightly increases nucleus-cell cross-correlations, and decreases the lag times; it lowers the correlation of nucleus-cell movements in U87 cells. Red lines crossing the plot indicate the control values. At least 10 cells per treatment from 3 independent experiments were analyzed. (TIF) [file pone.0093431.s007.tif]

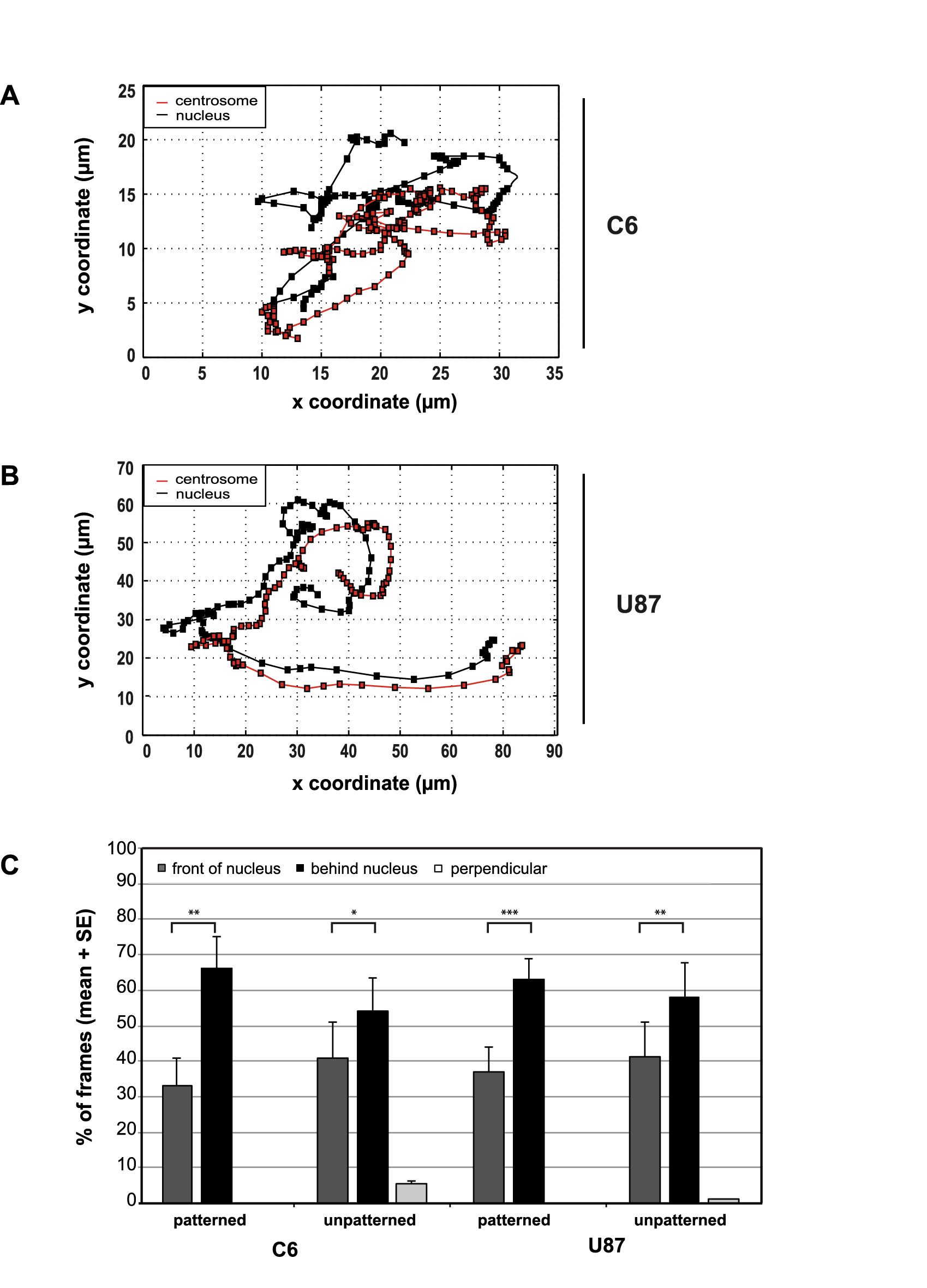

Supplement: Figure S8 — The centrosome is frequently behind the nucleus in cells migrating on patterned and non-patterned fibronectin surfaces. Centrosome (red marked lines) and nucleus trajectories (black marked lines) of representative C6 (A) and U87 cells (B) moving on homogenous fibronectin-coated surfaces (2D). (C) Centrosome positioning relative to the nucleus and the direction of the cell migration in C6 and U87 cells on patterned vs. non-patterned fibronectin surfaces. Note that the centrosome is most frequently localized behind the nucleus both in geometrically constrained (1D) and freely migrating (2D) cells. (TIF) [file pone.0093431.s008.tif]

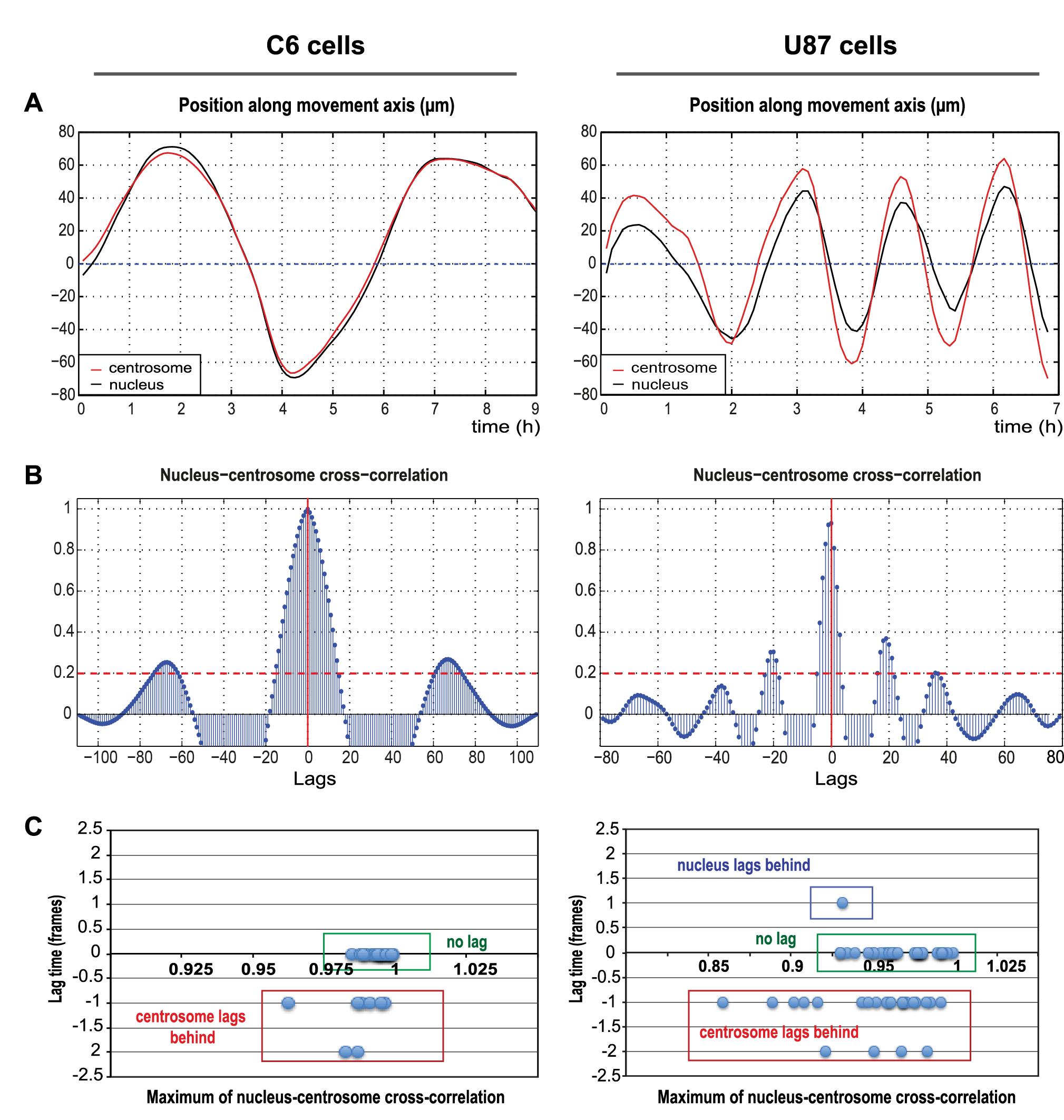

Supplement: Figure S9 — Confirmation of centrosome lagging by centrosome-nucleus positional cross-correlation analysis. Nucleus and centrosome positions of representative C6 and U87 cells (A) and their corresponding positional cross-correlation plots (B) illustrate the correlated movements of the nucleus and the centrosome in C6 and U87 cells. (C) Cross-correlation lags indicate that the centrosome either moves together or lags behind the nucleus in both cell lines. (TIF) [file pone.0093431.s009.tif]

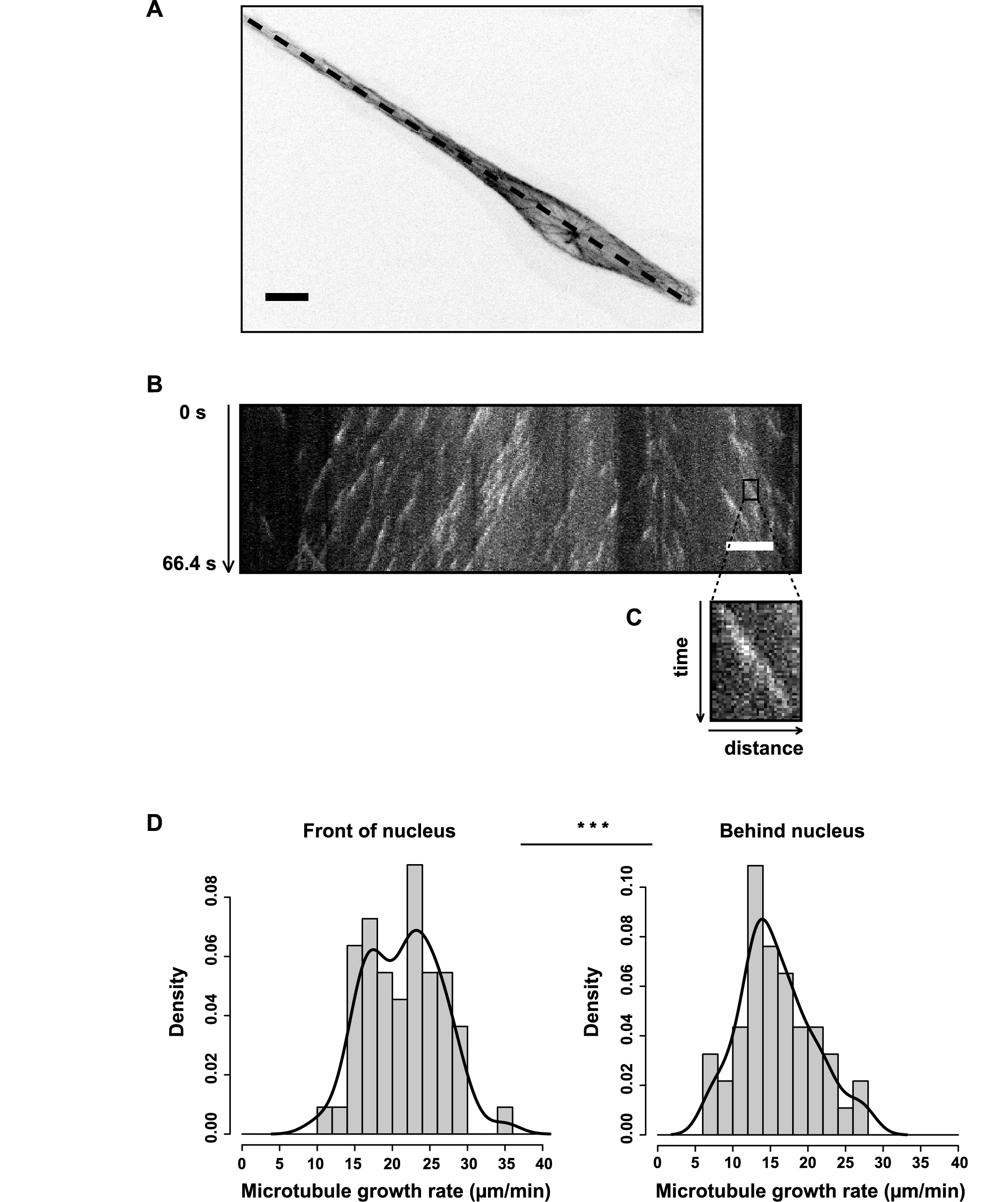

Supplement: Figure S10 — Microtubule dynamics of C6 cells revealed by YFP-EB3. C6 cells were transiently transfected with YFP-EB3, seeded on fibronectin patterns and imaged with a spinning disc microscope (speed: 400 ms/frame). To estimate the direction of nuclear movements, short (2 minutes) phase contrast time-lapse series of the selected cells were taken preceding YFP-EB3 imaging. (A) Inverted LUT (lookup table) image shows a C6 cell expressing the YFP-EB3 marker. This single image represents the maximum intensity projection of 50 time points. Scale bar: 10 μm. (B) Kymograph of YFP-EB3 along the dashed line marked in (A), indicating that there are more microtubules growing in front of the nucleus than behind. Scale bar: 5 μm. (C) Method for calculating microtubule growth rate. Speed (rate of microtubule polymerization) = travelled EB3 distance/time. (D) Histograms of microtubule growth in the direction of nuclear movement (front of the nucleus) and opposite of that measured within the same cells (n = 50 slopes of each direction, data obtained from 8 cells). Statistical analysis was performed using paired Wilcoxon test. Black lines overlaid on histograms indicate probability densities. (TIF) [file pone.0093431.s010.tif]
