# Supplementary material for: Nuclear Motility in Glioma Cells Reveals a Cell-Line Dependent Role of Various Cytoskeletal Components
Source: PLoS One. 2014 Apr 1;9(4):e93431. doi: 10.1371/journal.pone.0093431 (PMC3972233; doi:10.1371/journal.pone.0093431)
Supplement: Table S1 — Influence of cytoskeletal and motor protein inhibitors on oscillating cells. (DOCX) [file pone.0093431.s011.docx]

| **C6 cells** | **Control (n=176)** | **Blebbistatin 10 µM (n=92)** | **Cytochalasin D 100nM (n=33)** | **Nocodazole 10 nM (n=43)** | **Taxol 0.5 nM (n=12)** | **EHNA 0.5 mM (n=14)** |
| --- | --- | --- | --- | --- | --- | --- |
| **Average speed (median ± IQR)** | 34 ± 11 µm/h | 40 ± 15 µm/h | 33 ± 13 µm/h | 38 ± 9 µm/h | 27 ± 3 µm/h | 21 ± 7 µm/h |
| **Period length (median ± IQR)** | 7 ± 2 h | 7 ± 2 h | 8 ± 2.9 h | 8 ± 2.9 h | 8 ± 1 h | 7 ± 1 h |
| **Half peak amplitude (median ± IQR)** | 60 ± 23 µm | 69 ± 20 µm | 72 ±12 µm | 74 ± 14 µm | 50 ± 28 µm | 35 ± 27 µm |
| **U87 cells** | **Control (n=182)** | **Blebbistatin 10 µM (n=92)** | **Cytochalasin D 100nM (n=11)** | **Nocodazole 10 nM (n=27)** | **Taxol 0.5 nM (n=8)** | **EHNA 0.5 mM (n=26)** |
| **Average speed (median ± IQR)** | 57 ± 23 µm/h | 39 ± 18 µm/h | 28 ± 18 µm/h | 47 ± 16 µm/h | 54 ± 19 µm/h | 41 ± 15 µm/h |
| **Period length (median ± IQR)** | 2.4 ± 1 h | 3.5 ± 1.8 h | 6.2 ± 2 h | 2.8 ± 2.4 h | 5.1 ± 3.3 h | 2.9 ± 1.4 h |
| **Half peak amplitude (median ± IQR)** | 38 ± 9 µm | 38 ± 13 µm | 35 ± 17 µm | 47 ± 16 µm | 29 ± 18 µm | 15 ± 29 µm |
