# Supplementary figures and images for: Correction: Nuclear Motility in Glioma Cells Reveals a Cell-Line Dependent Role of Various Cytoskeletal Components
Source: PLoS One. 2014 Jul 2;9(7):e101593. doi: 10.1371/journal.pone.0101593 (PMC4079688; doi:10.1371/journal.pone.0101593)

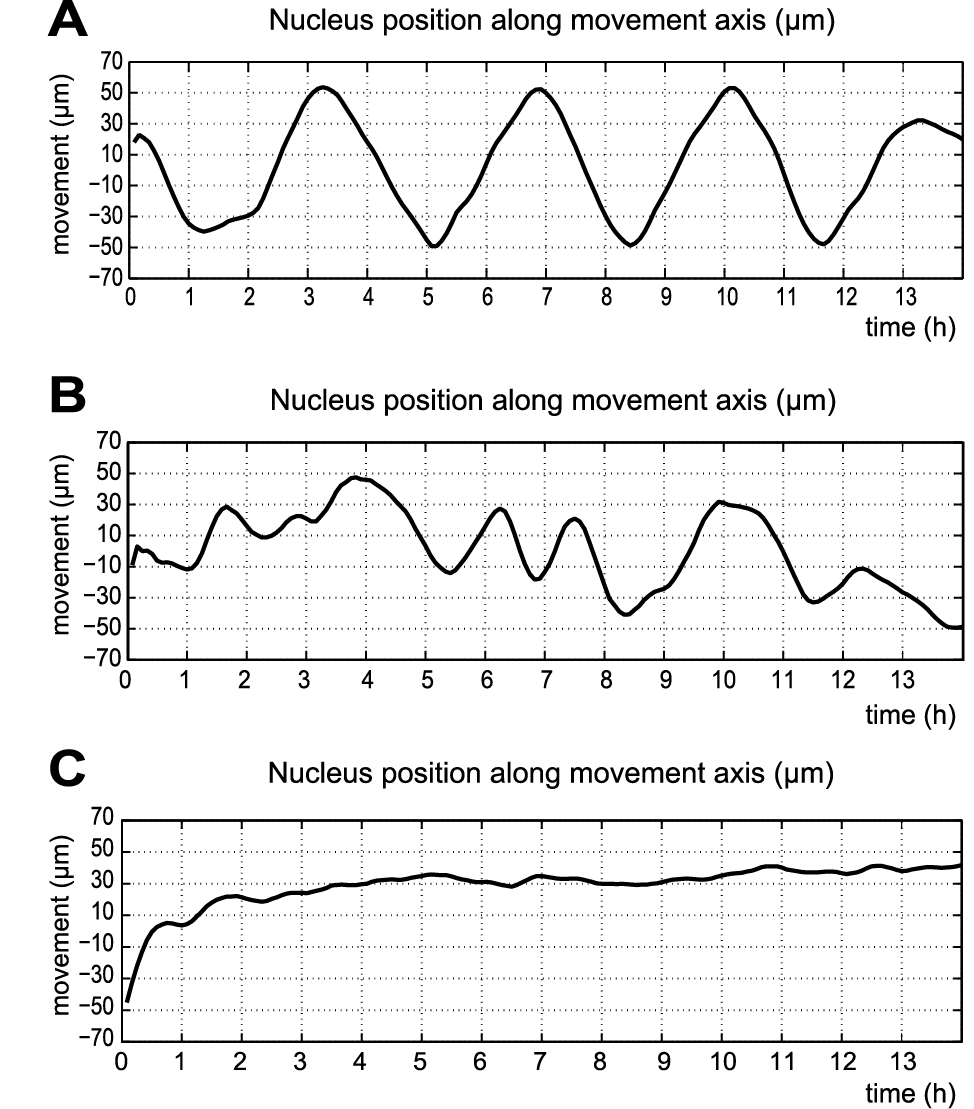

Supplement: Figure S2 — Coupling between nuclear migration and cellular movements. Cell extensions and nuclei of C6 and U87 cells seeded on patterns were manually tracked (n = 15). Representative example of an oscillating C6 (A) and U87 cell (B). Top panels: Positions of the cell center, the nucleus and the cell edges projected along the pattern over time. Middle panels: Relative position of the nucleus within the cell, normalized to the cell edges*. Allows visualizing the nuclear movements inside the cell. Lower panels: Related cross-correlation plots indicate no coupling between the movement of the nucleus and the cell centroid in C6 cells, and a strong correlation between their movements in U87 cells. Red vertical lines mark the lag at 0, red dashed lines indicate 95% confidence intervals. *Cell edges are defined at the start of tracking process, thus the “leading” or “trailing” edge terms are arbitrary. (TIF) [file pone.0101593.s001.tif]

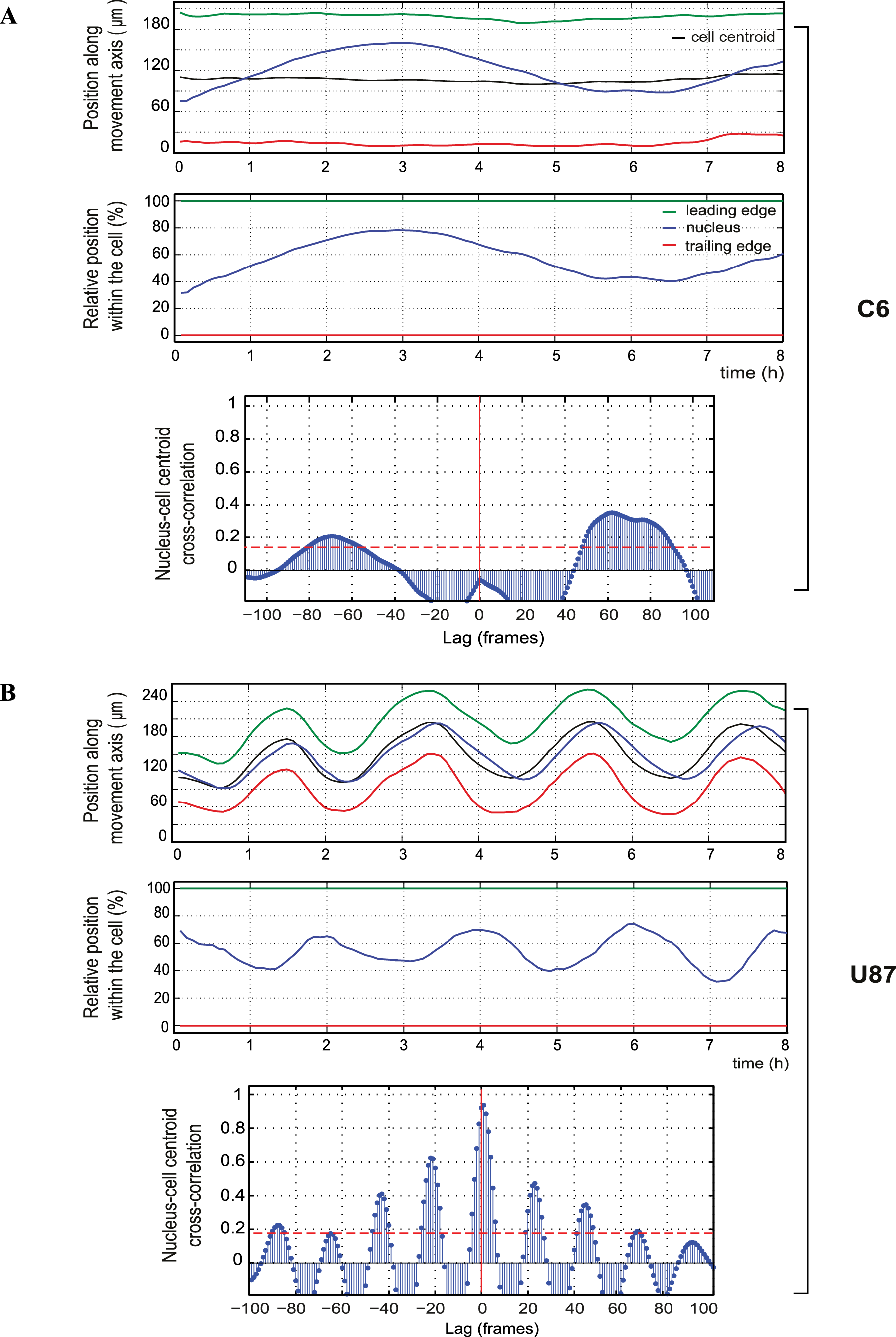

Supplement: Figure S3 — Categorization of nuclear movements in U87 cells. Based on the coordinates of nuclei projected to the movement axis (i.e. along the pattern) and visual inspection of their corresponding trajectories, we have established the following categories: (A) Oscillatory movement: nuclei display a periodic movement along the pattern in at least 80% of the measured time. (B) Irregular movement: nuclei move without recurrent periodicity. (C) No movement: nuclei show no significant positional change over most of the time. This means that the cumulative nuclear displacement within 14 hours was below 200 µm for C6 cells, or below 300 µm in the case of U87 cells. (TIF) [file pone.0101593.s002.tif]
